# Advancing Head and Neck Cancer Therapies: From Conventional Treatments to Emerging Strategies

**DOI:** 10.3390/biomedicines13051046

**Published:** 2025-04-25

**Authors:** Aleksandra Mordzińska-Rak, Ilona Telejko, Grzegorz Adamczuk, Tomasz Trombik, Andrzej Stepulak, Ewa Błaszczak

**Affiliations:** 1 Department of Biochemistry and Molecular Biology, Faculty of Medical Sciences, Medical University of Lublin, 1 Chodzki Street, 20-093 Lublin, Poland; 2 Independent Medical Biology Unit, Faculty of Pharmacy, Medical University of Lublin, 8b Jaczewski Street, 20-093 Lublin, Poland

**Keywords:** head and neck cancer, head and neck squamous cell carcinoma, conventional therapies, targeted therapy, immunotherapy, nanomedicine, nanoparticles, PROTAC

## Abstract

Head and neck cancers (HNCs), particularly head and neck squamous cell carcinoma (HNSCC), are among the most aggressive and prevalent malignancies of the upper aerodigestive tract. As the incidence of HNCs continues to rise, this cancer type presents a significant public health challenge. Despite conventional treatment options, such as surgery, chemotherapy, and radiotherapy, the five-year survival rates remain relatively low due to resistance to these therapies, local recurrence, local lymph node metastasis, and in some advanced cases also distant metastasis. Consequently, patients with HNCs face a high mortality risk and have reduced quality of life due to the side effects of chemo- and radiotherapy. Furthermore, targeted therapies and immunotherapies have also shown limited effectiveness in many cases, with issues related to resistance and the accessibility of these treatments. Therefore, new strategies, such as those based on combination therapies and nanotechnology, are being explored to improve the treatment of HNC patients. The proteolysis-targeting chimeras (PROTACs) also emerged as a promising therapeutic approach, though research is still ongoing to bring this technology into clinical practice. Here, we aim to highlight the current knowledge of HNC therapies, with a focus on recent advancements, including nanomedicine and PROTAC-based strategies. The development and advancement of novel emerging therapies hold promise for the improvement of patients’ survival and quality of life.

## 1. Introduction

**Figure 1:**
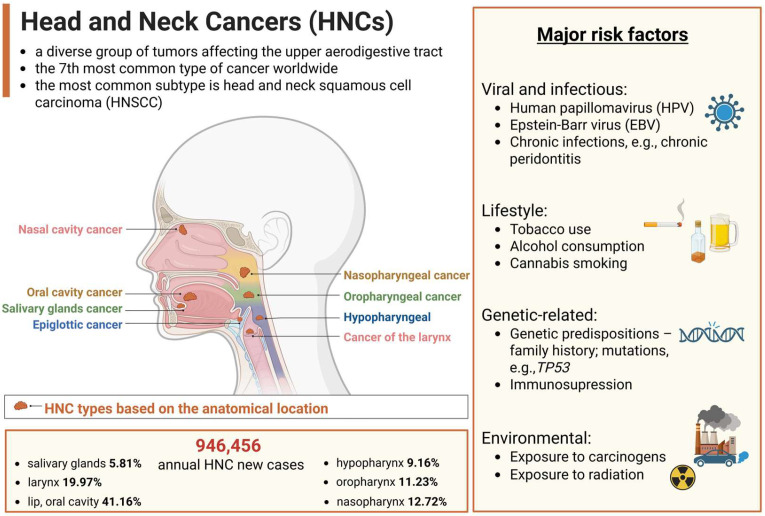
Overview of head and neck cancers (HNCs). The primary types of HNC based on their anatomical locations, and major risk factors, including tobacco and alcohol use, human papillomavirus (HPV) infection, or environmental exposures. Incidence data, representing annual new cases of HNC worldwide, based on global cancer statistics 2022 [4], with variations observed across different geographic regions. Created in BioRender. Blaszczak, E. (2025) (https://BioRender.com/).

Head and neck cancers (HNCs) comprise a heterogeneous group of tumors that arise in the anatomical sites of the upper aerodigestive tract (Figure 1). The predominant histological classification of HNCs is head and neck squamous cell carcinoma (HNSCC), accounting for approximately 90% of cases. HNSCC originates from the epithelial tissue lining the oral cavity, pharynx, and larynx [1,2]. These cancers can be categorized by HPV status (HPV-positive versus HPV-negative), which is associated with different biological and etiological characteristics. The major risk factor for HPV-positive tumors is the human papillomavirus (HPV) infection, while HPV-negative cancers are mainly associated with tobacco smoking and excessive alcohol intake [3]. HNCs remain a global health concern due to their high incidence and mortality with 946,456 new cases diagnosed and 482,001 deaths occurring each year worldwide [4]. Although treatment strategies have improved, the five-year survival rate for HNSCC remains relatively low, with fewer than 50% of patients surviving beyond this period [5]. Moreover, epidemiological studies estimate a 30% increase in HNC cases globally by 2030, highlighting the need for personalized treatment strategies [2] and the development of novel therapies to significantly improve patients’ quality of life.

While HNCs are a global health concern, affecting people in both developed and developing countries [6], trends vary by region and tumor subtype. For example, in Europe and in the USA, the high incidence rates are mainly driven by the increase in HPV-related oropharyngeal cancers (mainly HPV-16 and HPV-18) [7,8]. Population-based studies show a global trend of increasing HPV-positive HNC, while HPV-negative cases are declining in countries and regions like the USA, Canada, South Korea, and Hong Kong [9]. Over the next two decades, HPV-positive HNC is expected to become the dominant form, with oropharyngeal cancer surpassing oral cavity cancer in incidence across some European countries, e.g., the UK. As aforementioned, individuals with HPV-negative tumors tend to have a heavier cigarette smoking history, with their risk of mortality increasing with each additional pack-year, compared to HPV-positive cases. Interestingly, patients with HPV-positive tumors experience nearly a 60% lower risk of death after adjusting for factors such as age, ethnicity, cancer stage, smoking history, and treatment regimen [10]. This improved prognosis may be linked to fewer comorbidities in HPV-positive patients, enhanced anti-tumor immune responses, or the biological characteristics of HPV-positive tumors, such as greater radiosensitivity and lower mutational burden [11].

**Figure 2:**
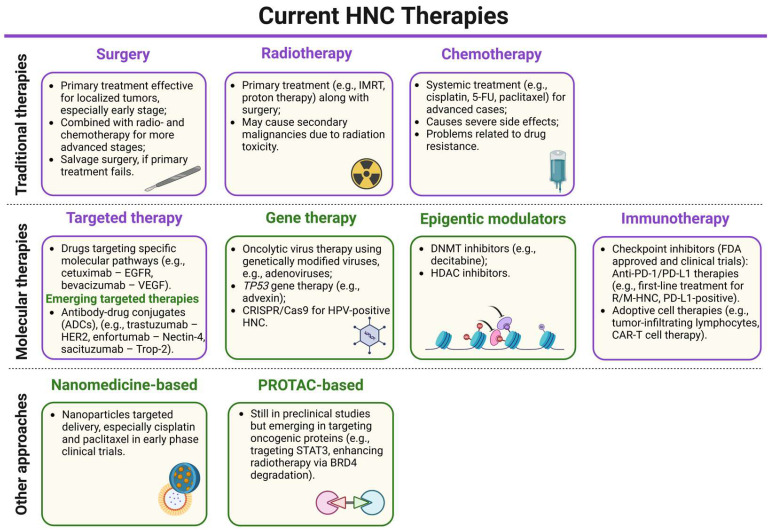
A schematic summary of the current head and neck cancer (HNC) therapies (detailed description further in the text). The current treatments are marked in purple boxes, while the emerging therapies are in green boxes. For most HNC patients, depending on the clinical status, the standard treatment involves single or mixed approaches of surgery, RT, or chemotherapy to reduce recurrence risk. Targeted therapy is also used as it exhibits fewer side effects and higher specificity, though not all patients respond to it. Similarly, immunotherapy targeting programmed cell death 1 (PD-1) and programmed death-ligand 1 (PD-L1) therapy is a first-line treatment for recurrent or metastatic PD-L1-positive tumors. The main emerging therapeutic strategies in preclinical or clinical trials include immune checkpoint inhibitors (ICIs), antibody–drug conjugates, small molecule inhibitors, epigenetic modulators, and oncolytic virus therapies. Additionally, nanotechnology-based approaches and PROTAC-based approaches are also emerging treatments for these cancers. IMRT—intensity-modulated radiotherapy, 5-FU—5-fluorouracil, EGFR—epidermal growth factor receptor, VEGF—vascular endothelial growth factor, ADC—antibody–drug conjugate, HER2—human epidermal growth factor receptor 2, Trop-2—trophoblast cell surface antigen 2, CRISPR/Cas9—clustered regularly interspaced short palindromic repeats/CRISPR-associated protein 9, DNMT—DNA methyltransferase, HDAC—histone deacetylase, R/M-HNC—recurrent or metastatic head and neck cancer, CAR-T cell—chimeric antigen receptor T cell, STAT3—signal transducer and activator of transcription 3, BRD4—bromodomain-containing protein 4. Created in BioRender. Blaszczak, E. (2025) (https://BioRender.com/).

One of the key challenges in managing HNCs is that they are often diagnosed at advanced stages, reducing the effectiveness of surgical treatments and leading to poorer patient prognosis with lower survival rates. Cisplatin (CDDP) is a widely used chemotherapeutic agent for HNC, often administered as adjuvant therapy for locally advanced cases or combined with radiotherapy (RT) in the so-called chemoradiotherapy (CRT) to improve treatment efficacy. CDDP monotherapy has an efficacy rate of around 40% [12]. Long-term follow-up of a trial by Cooper et al. [13] showed no significant improvement in overall survival (OS) from adding CDDP to postoperative RT in high-risk resected HNC patients, with 10-year OS rates of 27.0% for RT alone and 29.1% for RT plus cisplatin (*p* = 0.31), emphasizing the need for more effective strategies in select high-risk groups. This is especially true as CRT can lead to substantial side effects and negatively impact patients’ quality of life [14]. Two immune checkpoint inhibitors (ICIs), nivolumab and pembrolizumab, were approved by the Food and Drug Administration (FDA) for treating recurrent or metastatic HNSCC (R/M-HNSCC), with pembrolizumab serving as the first-line therapy for unresectable tumors, significantly increasing the OS of R/M-HNC [15,16,17]. However, the effectiveness of these therapies is often limited by factors such as immune resistance, the lack of long-term responses in some patients, and treatment toxicities [18]. Therefore, there is a constant need for novel treatment development. In this review, we summarize the current knowledge of HNC therapeutic strategies (Figure 2). We discuss the advantages and limitations of the currently used treatments. Moreover, we emphasize emerging technologies for treating this heterogeneous cancer type, including targeted therapies, immunotherapies, nanotechnologies, and PROTAC technology.

## 2. Conventional Treatment Options for HNCs

The choice of treatment for HNC depends on the disease stage, tumor location (anatomical site), and feasibility of surgical intervention. It typically involves surgery, RT, chemotherapy, or a combination of surgery with RT or chemo- and radiotherapy. For early-stage HNC (stage I or II), around 30–40% of patients are treated with either surgery or RT, leading to improved survival outcomes. In contrast, more than 60% of patients with advanced-stage HNC (stage III or IV) require more advanced therapeutic approaches [19,20].

### 2.1. Surgery

HNC treatments typically involve surgical intervention, followed by postoperative radiation or chemotherapy, if required. Surgery is usually the primary treatment for oral carcinomas. In more advanced stages, treatment may include postoperative radiation, chemoradiation, targeted therapy for oncogenes, and immunotherapy [21,22]. Surgical approaches for treating most HNCs include open procedures, endoscopic methods, and robotic surgery. The goal is to remove enough tumor tissue to reduce the risk of local and regional recurrences, which can impact long-term patient’s survival. A 1 cm margin of three-dimensional dissection is considered adequate for oral carcinoma surgery. However, larger margins may lead to aesthetic and functional complications. Surgical effectiveness is mainly influenced by the cancer’s stage at diagnosis, with earlier-stage cancers showing better responses [1,3].

After excising the primary tumor, reconstructive surgery is typically necessary to restore both the function of the oral cavity and the appearance of the head and neck. The choice of reconstruction method depends on factors such as the defect characteristics, the patient’s medical history, the expertise of the surgeon, and the prognosis. Reconstructive procedures generally follow the so-called ‘reconstructive ladder’, starting with skin grafts and progressing to microvascular free flaps, with unrestricted tissue transfers being a reliable and commonly used technique for oral reconstruction [21]. Microvascular free flaps have become the standard for reconstructing tissue defects in advanced HNC cases. However, complications are frequent, affecting more than 70% of patients [23], particularly those who are elderly, have multiple comorbidities, and are heavy alcohol and tobacco users, which increases the likelihood of both surgical and medical complications [24].

### 2.2. Radiotherapy

RT in combination with surgery is a primary treatment option for HNC. RT doses typically range from 54 to 70 Gy (1 Gray corresponds to the absorption of 1 joule of radiation energy per kilogram of tissue), delivered through a standard fractionation schedule of 2 Gy per fraction, one fraction per day, five days a week. In high-risk cases, the standard nonsurgical treatment approach involves combining RT with concurrent cisplatin (100 mg/m^2^ every three weeks) [25]. Currently, the most widely used form of RT is external beam photon therapy [26]. To improve local tumor control, while minimizing RT toxicity, this approach is evolving toward a more personalized treatment. Technological advancements in photon-based RT have significantly enhanced dose distribution conformity for HNC patients [27]. Moreover, the introduction of heavy particle radiation (hadron therapy) [28], and importantly, a deeper understanding of the interaction between photons and the immune response of both the tumor and the host, have opened up new treatment opportunities [29]. Despite technological improvements, RT can cause acute and late toxicities [30], significantly diminishing the quality of life for HNC patients. Furthermore, some HNC tumors develop radioresistance, leading to recurrence and treatment failure. This can be related to tumor hypoxia, changes in gene expression, and induced DNA repair mechanisms [31]. The heterogeneity of these tumors may also play a considerable role. Additionally, RT shows limited effectiveness in advanced cases, particularly metastatic HNCs, and in such advanced cases, occurring radioresistance leads to treatment failure [32].

### 2.3. Chemotherapy

Chemotherapy plays a crucial role in the palliative treatment of HNCs and it is also used before the main treatment (neoadjuvant therapy) to help decrease the tumor size, thus improving the chances of preserving the larynx [33]. It is now widely accepted that in HNC patients diagnosed with stage III or IV of tumors, the standard regimen of chemotherapy includes CDDP, 5-fluorouracil (5-FU), and docetaxel or paclitaxel [20]. Cisplatin demonstrated high response rates (RRs) and improved OS compared to best supportive care and initially used methotrexate, establishing its role in recurrent or metastatic head and neck cancer (R/M-HNC) treatment [34]. The combination of CDDP with 5-FU became the standard regimen due to its enhanced RRs and manageable toxicity, despite not significantly prolonging the OS [35,36]. Recent studies have shown that patients with p16-negative HNC (the lack of p16 protein expression, a biomarker for HPV infection) who missed weekly CDDP cycles had significantly worse OS and progression-free survival (PFS) compared to those who received seven to eight cycles of treatment with CDDP. In contrast, there was no significant difference in OS between patients with p16-positive tumors (elevated levels of p16 protein expression) who missed cisplatin cycles and those who completed the full regimen. Cytopenia, a reduction in the number of blood cells, was the most common reason for treatment interruption. These findings suggest the importance of adherence to cisplatin cycles, especially for p16-negative tumors, in improving patient outcomes [37].

Another drug, docetaxel, which inhibits microtubule depolymerization and disrupts mitosis, demonstrated significant effectiveness as a standalone treatment for both recurrent and advanced HNCs [38,39]. Similarly, paclitaxel, also an FDA-approved chemotherapeutic agent, is used in combination with other chemotherapeutic compounds, e.g., CDDP and carboplatin, and shows less toxicity than cisplatin alone [40]. The combination of biological and chemotherapeutic agents with RT has shown strong effectiveness in improving local tumor control and enhancing patient survival outcomes [41]. For patients with recurrent or metastatic tumors, therapeutic options include immune checkpoint inhibitors, platinum derivatives, 5-FU, and cetuximab [42]. A retrospective analysis evaluated the efficacy and tolerability of paclitaxel, carboplatin, and cetuximab (PCC) in HNC patients. Among 58 patients with R/M disease, the RR was 22%, with a mean PFS of 7.1 months and OS of 15.2 months. In the induction cohort (*N* = 22), 86% successfully completed treatment, with an RR of 64%. In 52% of patients, ≥grade 3 toxicities appeared, though PCC was well-tolerated and showed promising PFS/OS outcomes in R/M disease [42].

### 2.4. Limitations of Conventional HNC Treatments

To sum up, conventional treatments for HNCs, including surgery, radio-, and chemotherapy, have several significant disadvantages. Surgery, particularly when large portions of tissue are removed, can lead to functional or cosmetic impairments, affecting speech, swallowing, and facial appearance [43,44]. RT can cause side effects such as dry mouth, difficulty swallowing, inflammation of the mucous membranes, and long-term damage to healthy tissues, all of which can impact treatment and recovery [14,45]. Patients undergoing radio- and chemotherapy often experience a range of side effects, including speech disorder, dysphagia, pain, and depression, which can significantly affect their quality of life. Xerostomia (‘dry mouth’) is also a major complication affecting patients undergoing radiation or chemoradiation [46]. Apart from side effects such as systemic toxicities, these therapies are often less effective in advanced stages of HNC, where tumors may metastasize or become resistant to treatment.

## 3. Emerging Therapies in HNC

Due to the high heterogeneity of HNC, side effects of standard therapies, and the increasing incidence of the disease, there is a greater need for the development of new therapies. Numerous clinical trials involving patients with HNC, particularly HNSCC, have been conducted globally according to the clinical trials database (URL: https://clinicaltrials.gov, accessed on 2 March 2025). While searching for studies related to HNSCC (specified search term; start date up until 2 March 2025), 1777 were found. Out of these, 577 trials have been completed, while 436 are still in the recruitment phase, categorized as recruiting, 198 active but not recruiting, 219 terminated, 95 not yet recruiting, 4 enrolling by invitation, 7 suspended, 71 withdrawn, and 169 were found as unknown. Based on the information from the Clinicaltrials.gov database, we present some of the selected compounds that were either approved by the FDA for the treatment of HNCs or that are undergoing clinical trials (Table 1).

### 3.1. Targeted Therapy

**Figure 3:**
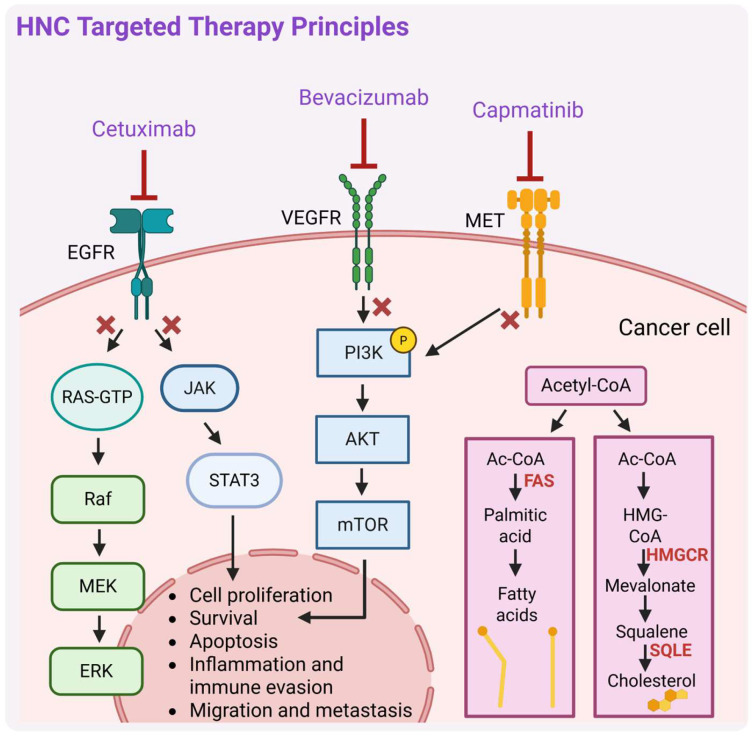
A schematic representation of the principal mechanisms of targeted therapy in head and neck cancers (HNCs). RAS-GTP—Rat sarcoma virus oncogene-guanosine triphosphate, Raf—rapidly accelerated fibrosarcoma, MEK—mitogen-activated protein kinase, ERK—extracellular signal-regulated kinase, JAK—Janus kinase, PI3K—phosphatidylinositol 3-kinase, AKT—serine/threonine kinase, mTOR—mechanistic target of rapamycin, MET—mesenchymal–epithelial transition factor, FAS—fatty acid synthase, HMG-CoA—3-hydroxy-3-methylglutaryl coenzyme A, HMGCR—HMG-CoA reductase, SQLE—squalene epoxidase. Description in text. Created in BioRender. Blaszczak, E. (2025) (https://BioRender.com/).

Targeted therapies (Figure 3) have significantly advanced HNC treatment by precisely ‘attacking’ cancer cells while minimizing toxicity. A key breakthrough was the identification of EGFR, as a driver of tumor growth and metastasis, and its expression in HNC [54,55]. Hence, the idea of targeting it emerged. One of the monoclonal antibody-based drugs targeting EGFR, cetuximab’s efficacy was evaluated in combination with RT for HNC patients, demonstrating enhanced RT effectiveness and its significant role as a ‘radiosensitizer’, leading to FDA approval of such therapy for locally advanced HNSCC [32,47,56]. It was also shown to exhibit dose-dependent pharmacokinetics, tolerability, and biological activity when combined with cisplatin in patients with advanced EGFR-overexpressing tumors [57]. Combining cetuximab with platinum-based chemotherapy (CDDP, carboplatin) plus 5-FU extended median OS from 7.4 months (chemotherapy alone group) to 10.1 months (group receiving platinum-5-FU plus cetuximab), establishing combination therapy with cetuximab as an effective treatment for platinum-resistant R/M-HNC [48], even though its efficacy compared to CRT was debated with mixed results from subsequent trials. Cetuximab combined with RT demonstrated better outcomes over RT alone but the trial did not include clinical data on cetuximab with RT in comparison to CRT [47]. A subsequent trial showed that cetuximab plus RT was just as effective as CRT after induction chemotherapy [58], while another trial suggested that it might be less effective, though the study was not specifically designed to confirm this with statistical significance [59]. Overall, cetuximab is better established for R/M-HNC than for locally advanced cases, but its benefits must be weighed against its side effects [60]. In the pivotal clinical trial by Bonner et al. [47], cetuximab did not worsen the typical side effects of RT in HNSCC, such as mucositis, xerostomia, or dysphagia. However, during routine clinical practice, a greater frequency of severe radiation-related dermatitis than in the Bonner trial was observed [61,62]. Cetuximab and radiation caused a usually uncommon severe skin rash of Grade 3 in a 61-year-old man with oropharyngeal cancer [63]. Moreover, other side effects were reported, such as anaphylaxis [64], and respiratory-related (i.e., acute eosinophilic pneumonia) [65] and hematologic toxicity [66,67]. More recent retrospective studies based on data from the FDA Adverse Event Reporting System (FAERS) reported that cetuximab typically affects the blood and lymphatic systems and is associated with more serious outcomes with multiple medications (polytherapy) compared to cetuximab alone. Additionally, more serious outcomes concerned the therapy with cetuximab in countries outside the US than within the US [68]. Despite cetuximab’s efficacy and safety improvements in HNC therapy, further advancements are needed to enhance clinical outcomes and improve quality of life [32,69] through more precise therapeutic strategies and reduced treatment-related toxicities.

Currently, there are still several clinical trials that investigate cetuximab therapy in combination with agents targeting different effectors, including immune checkpoints, e.g., programmed cell death 1 (PD-1) and programmed death-ligand 1 (PD-L1) inhibitors, vascular endothelial growth factor/vascular endothelial growth factor receptor (VEGF/VEGFR) inhibitors [32], and tyrosine kinase inhibitors, e.g., MET inhibitors. Active, ongoing trials are assessing the efficacy and safety of cetuximab in combination with an anti-PD-L1 inhibitor avelumab in advanced HNC (NCT03494322; phase II [70]), or both drugs administered after three cycles of platinum and taxane-based chemotherapy in patients with R/M-HNSCC (NCT06869473). For R/M-HNSCC, cetuximab is also being evaluated in combination with the PD-1 inhibitor nivolumab (NCT03370276; phase I/II). Overall, combining cetuximab with nivolumab is showing significant anti-cancer effects, while having manageable side effects in patients with R/M-HNSCC, regardless of their previous treatment regimens [71]. Current clinical development is also evaluating the MET tyrosine inhibitor capmatinib, initially approved by the FDA in 2020 for the treatment of metastatic non-small-cell lung cancer (NSCLC; particularly tumors with a mutation that leads to MET exon 14 skipping), for potent use in HNC cancer therapy [72].

The addition of bevacizumab, a monoclonal antibody that targets vascular endothelial growth factor (VEGF), a potent inducer of angiogenesis, to chemotherapy in R/M-HNSCC improved PFS (6.0 vs. 4.3 months, *p* = 0.0014) and RRs (35.5% vs. 24.5%, *p* = 0.016) but did not significantly improve OS (*p* = 0.22). Increased toxicities were observed with bevacizumab, including a higher rate of grade 3 to 5 bleeding events (6.7% vs. 0.5%, *p* < 0.001) and treatment-related deaths (9.3% vs. 3.5%, *p* = 0.022). The findings highlight the need for biomarker-driven studies to identify patients who may benefit from angiogenesis inhibitors with improved safety profiles [73].

Recently, studies have also focused on targeted therapies involving the modulation of lipid metabolism, which plays an important role in tumor progression and drug resistance. For instance, inhibition of squalene epoxidase (SQLE), a key enzyme in endogenous cholesterol synthesis, increased squalene accumulation and enhanced the sensitivity of HNSCC cells to EZH2 (enhancer of zeste homolog 2; histone methyltransferase) inhibitors. Notably, EZH2 inhibitors strongly induce the expression of genes involved in the cholesterol synthesis pathway. Targeting EZH2 alone may not be enough to effectively treat solid tumors due to metabolic adaptations; however, combining it with SQLE inhibition shows promise in overcoming these limitations [74].

Another potentially significant targeting approach in HNC can be targeting fatty acid-binding proteins (FABPs). Overall, FABPs facilitate the transport and metabolism of long-chain fatty acids, influencing key cellular processes such as proliferation, survival, and inflammation [75]. FABP5 is significantly overexpressed in oral squamous cell carcinoma (OSCC), and it is linked to tumor progression and invasiveness. Inhibiting FABP5 disrupts lipid signaling pathways essential for tumor cell growth, potentially impairing cancer progression [76,77]. While specific inhibitors are still under investigation, early research suggests that blocking FABP5 function could reduce cell viability and metastatic rate, highlighting its potential for targeted therapy in HNC.

A promising therapeutic target related to lipid metabolism is also fatty acid synthase (FAS, encoded by FASN gene), which catalyzes the endogenous synthesis of saturated fatty acids from acetyl-CoA and malonyl-CoA and regulates key processes such as cell growth and proliferation [78]. Given its involvement in tumor progression, FAS has emerged as a promising therapeutic target also in HNCs. Several FAS inhibitors, both natural and synthetic, have demonstrated anticancer activity by disrupting tumor cells’ lipogenic dependence with the FDA-approved drug orlistat (used for the treatment of obesity) being an example [79]. However, more research is needed to develop specific inhibitors and further potential of FAS-targeted therapies in HNC treatment. Current research is also ongoing to investigate compounds targeting metabolic reprogramming pathways in combination with immunotherapy (immunotherapy is further described in the next section), e.g., ICIs, which have the potential to counteract tumor progression and immune suppression induced by the metabolic switch in many cancers [80]. Here, however, further research is still needed to validate the safety, efficacy, and specificity of such treatments.

### 3.2. Immunotherapy

**Figure 4:**
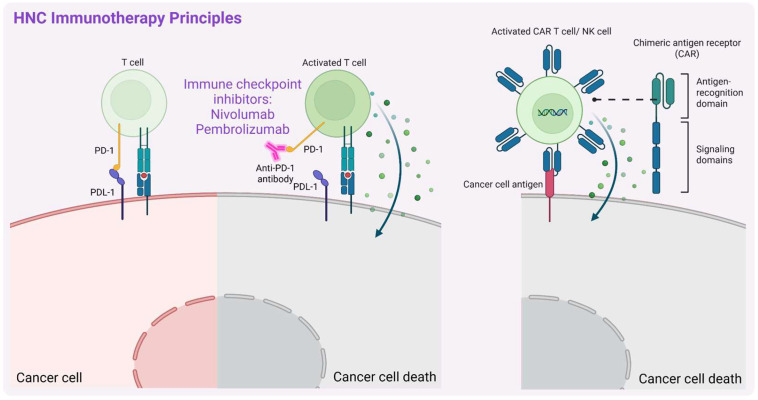
A schematic representation of the principal mechanisms of immunotherapy in head and neck cancers (HNCs). Description in text. Created in BioRender. Blaszczak, E. (2025) (https://BioRender.com/).

HNC has one of the most pro-inflammatory tumor microenvironments (TMEs) among solid tumors [81,82], making immunotherapy (Figure 4) a promising therapeutic approach. This strategy enhances the body’s immune response against tumors, and in the context of HNCs is currently being explored, especially based on ICIs, e.g., via targeting PD-1/PD-L1 or CTLA-4 (cytotoxic T-lymphocyte-associated protein 4) and adoptive cell therapies, e.g., tumor-infiltrating lymphocytes or chimeric antigen receptor T cell (CAR-T cell) therapy. ICIs have emerged as a promising treatment, especially for R/M-HNSCC. Clinical trials have demonstrated the efficacy of anti-PD-1 antibodies, such as nivolumab and pembrolizumab, leading to FDA approval for their use in advanced-stage disease [15,16,17]. However, a relatively modest durable response rate of 15–20% was reported [83], highlighting the need for combination strategies and further research to optimize their integration into an effective HNSCC treatment. Additionally, some patients experience immune-related side effects, such as dermatitis, colitis, hepatitis, and pneumonitis, which may require pausing ICI therapy or considering alternative treatments [84,85].

Hence, other immunotherapy-based approaches are still being developed. For example, durvalumab, a high-affinity humanized monoclonal antibody targeting PD-L1 (blocking the PD-1/PD-L1 pathway), demonstrated notable efficacy and a good safety profile in phase I/II clinical trial (NCT01693562, all data referring to the trials with an ID indicated were retrieved from: https://clinicaltrials.gov, unless stated otherwise). Durvalumab (MEDI4736) also shows good antitumor activity and safety as monotherapy in R/M-HNSCC PD-L1-positive patients (NCT02207530). However, neither durvalumab alone nor in combination with tremelimumab, a monoclonal antibody that targets CTLA-4, significantly improved patients’ OS (NCT02369874). Another phase III clinical trial (NCT04146402) evaluated the efficacy and safety of finotonlimab (SCT-I10A), a PD-1 monoclonal antibody, combined with cisplatin plus 5-FU (abbreviated further in combination as C5F) as a first-line treatment for R/M-HNSCC in an Asian population. Among 370 patients, those receiving finotonlimab plus C5F had a median OS of 14.1 months, significantly longer than the 10.5 months observed in the placebo plus C5F group (hazard ratio (HR) = 0.73, *p* = 0.0165), confirming the survival benefit and manageable safety profile of finotonlimab plus C5F and supporting its use as a first-line therapy for R/M HNSCC [86].

Interestingly, statins have recently been shown to enhance the efficacy of PD-1 immune checkpoint blockade in HNSCC. Statins are routinely administered medications for lowering cholesterol levels, particularly in the treatment of hypercholesterolemia [87,88]. Additionally, they exhibit anticancer, anti-inflammatory and immunomodulatory effects [89,90]. Statins have not been clinically used in the treatment of patients with HNC; however, several studies have reported a protective role of statins in HNC progression and a reduction in both total and disease-related mortality in association with statin use (reviewed in [88]). Ex vivo co-culture assays of murine cancer cells and tumor-infiltrating lymphocytes showed that all seven tested statins inhibited tumor cell proliferation, while simvastatin and lovastatin also enhanced T cell-mediated tumor killing. In syngeneic mouse models (MOC1 and TC-1; mouse oral cancer), daily oral administration of these statins improved tumor control and survival when combined with PD-1 blockade, with lovastatin achieving MOC1 tumor rejection in 30% of cases. Mechanistic analyses suggest that statins promote T cell activation and shift macrophage polarization from M2 toward a pro-inflammatory M1 phenotype [91], possibly by reducing cholesterol levels in the TME, which is known to have an immunosuppressive effect [92,93,94]. Their role needs to be further investigated in this context, though this supports their use as cost-effective drugs to enhance immunotherapy in HNC.

Cancer immunotherapy is also challenged by inherent limitations, including the development of resistance due to various mechanisms. A recent study by Zhang et al. [95] identified integrin subunit beta 6 (ITGB6) as a key mediator of resistance to anti-CD276 therapy with enoblituzumab in HNSCC using a murine model. ITGB6 regulates the expression of the chemokine ligand CX3CL1, which recruits PF4+ macrophages that suppress cytotoxic CXCR6+ CD8+ T cells, reducing treatment efficacy. Blocking the CX3CL1–CX3CR1 axis restores sensitivity to anti-CD276 treatment. What is more, inhibition of ITGB6 resensitizes mice resistant to anti-PD1 treatment offering new strategies to overcome resistance in cancer immunotherapy [95].

To overcome issues related to resistance and other treatments, new immunotherapy strategies are also being developed for HNC. Among these, CAR-T cell therapy takes advantage of the fact that it functions independently of major histocompatibility complex (MHC)-associated antigen presentation [96], allowing it to bypass one of the major immune evasion mechanisms used by cancer cells. Unlike conventional immunotherapies that rely on MHC expression for antigen recognition, CAR-T cells can directly target tumor-associated antigens, making them effective even in tumors with impaired MHC presentation [97]. This feature makes CAR-T cell therapy particularly promising for HNSCC, where immune evasion is a significant challenge. So far, however, the transformation of this therapy into clinical practice is also a challenge due to its limited effectiveness because of tumor heterogeneity, immunosuppressive TME, and off-target toxicity [96]. Hence, potentially safer alternatives based on the chimeric antigen receptor natural killer cells (CAR-NK cells) are being investigated. Another recent study by Manzar et al. [98] showed that RT enhanced the effectiveness of CD70-targeting via CAR-NK cells in HNSCC treatment by upregulating CD70 expression in tumor cells. This radiosensitization effect persisted even after CD70 levels returned to the baseline, and the study provided the first preclinical evidence of synergy between RT and CAR-NK cell therapy in solid tumors [98]. Nowak et al. [99] demonstrated the potential of CAR-NK-92 cells targeting HER1/EGFR in treating HNSCC. Anti-HER1 CAR-NK-92 cells effectively eliminated HNSCC cells (including those derived from representative HNSCC cell line models and patient-derived primary cell lines) in 2D and 3D spheroid co-culture experiments. This also enhances NK cell degranulation and interferon-gamma (IFNγ) secretion while inducing target cells’ apoptosis. Additionally, the remaining patient-derived primary HNSCC cells exhibited increased expression levels of the potential cancer stem cell marker CD44v6 [99]. In line with this, another study demonstrated that anti-CD44v6 CAR-NK cells showed enhanced cytotoxicity against HNSCC cell lines, including those with both high and low CD44v6 expression, suggesting their potential as a promising therapeutic option [100]. Further preclinical trials are, however, necessary to optimize these treatment strategies.

Over the past few years, immunotherapy has explored not only checkpoint inhibitors but also other antibody-based molecules for their clinical use in the treatment of different cancers. One group consists of antibody–drug conjugates (ADCs), in which a cytotoxic payload is attached to an antibody recognizing a tumor-specific or tumor-associated antigen (TSA or TAA, respectively). Several of them have been tested in clinical studies in patients with HNSCC with a promising outcome (recently summarized in a systematic review [101] for HNSCC and nasopharyngeal carcinoma). Others represent molecules with multiple specificities, targeting tumor cells via TSAs or TAAs on the one hand and engaging the immune system on the other hand. For instance, the so-called Bispecific T-cell engagers (BiTEs) bind to TAAs and a protein complex subunit CD3 epsilon on T-cells resulting in T-cell activation, cytokine release, and secretion of perforin and granzymes leading to the tumor cell death (recently reviewed in [102]). There are also other bi-specific antibodies (BsAbs) that, for example, block immunosuppression by the simultaneous binding to immune checkpoints on the tumor cells and T-cells. This leads to the disruption of T-cell exhaustion signals and sustains anti-tumor activity. Several of these molecules have also been clinically tested in HNSCC patients (recently summarized in [101]). Interestingly, multiple NK-engagers such as TriKE [103,104,105] and tetraspecific ANKET [106] have been developed and are under evaluation. Clinical trials should consider side-effects of such molecules; for example, ADCs have been reported to cause myelosuppression and a pneumonitis case, while BsAbs caused infusion-related reactions [101]. Hence, further research is needed to advance the design of such molecules in the context of their improved efficacy and reduced toxicity.

Another approach in HNSCC therapy involves the use of tumor-infiltrating lymphocyte TILs adoptive therapy. This therapy is based on the isolation of TILs from the tumor, their ex vivo expansion, and reinfusion into the patient. It has been clinically evaluated in several cancer types including melanoma, NSCLC, and breast cancer, but also HNSCC with a significant response rate, efficacy, and an acceptable safety profile [107,108,109,110]. This approach also requires further investigation.

### 3.3. Nanomedicine in HNC Treatment

**Figure 5:**
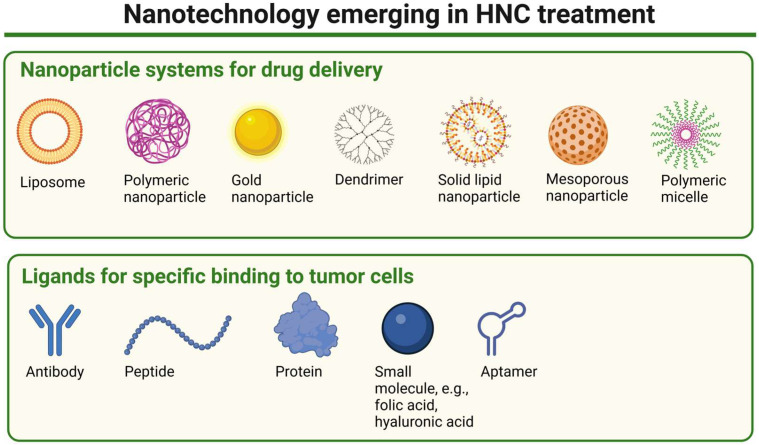
A schematic representation of nanoparticle types emerging in HNC treatment and examples of ligands potentially used to enhance specific binding to tumor cells. Created in BioRender. Blaszczak, E. (2025) (https://BioRender.com/).

The use of nanotechnology holds promise in oncology, particularly in the development of therapeutic delivery systems and novel, more effective drugs. It is also important in enhancing the efficacy of HNC treatments while minimizing side effects. By enabling targeted delivery of therapeutic agents, it can improve drug accumulation in tumor tissues and reduce systemic toxicity and side effects [111,112,113]. One of its key applications involves the use of nanoparticles (NPs) (Figure 5), which can be employed through two primary targeting strategies: passive and active targeting. Passive targeting takes advantage of the unique physiological characteristics of tumors, such as leaky vasculature and impaired lymphatic drainage, to facilitate the accumulation of NPs at the tumor site (both primary tumor and metastatic sites) [114,115]. The so-called enhanced permeability and retention (EPR) effect allows nanosystems and macromolecules larger than 40 kDa to penetrate the tumor stroma. In contrast, small-molecule drugs are rapidly cleared from the bloodstream due to their short circulation time. By encapsulating therapeutic agents within NPs, passive targeting prolongs systemic circulation, enhances tumor selectivity, and reduces off-target effects, improving overall treatment efficacy. This approach is, however, not devoid of disadvantages. The efficacy of passive targeting is often limited by factors such as tumor heterogeneity, high interstitial pressure, abnormal vascularization, and incomplete accumulation compared to normal tissues (increased by 20–30% only) [116]. Another strategy of targeting, active targeting, also referred to as ligand-mediated targeting, involves functionalizing nanocarriers with ligands that selectively bind to overexpressed receptors on tumor cells, improving drug accumulation while minimizing off-target effects. These specific ligands include antibodies, proteins, peptides, sugars, or small molecules (e.g., folic acid, hyaluronic acid). Such an approach enables selective binding to cancer cells as ligand–receptor interactions facilitate internalization, enhancing the precision and efficacy of NP-based therapies. The cost of production of such nanocarriers is, however, high, and more importantly, in the case of some (e.g., with antibodies used as ligands), potential immunogenicity is also a limitation [116,117,118].

There is a growing number of both preclinical and clinical studies exploring the use of NPs in the treatment of various cancers, including HNCs. These investigations primarily focus on different classes of NPs, such as metallic (primarily gold NPs), metal oxides, polymeric, silica-based, and liposomal nanoparticles (recently summarized by [119]) to enhance therapeutic efficacy and reduce the toxicity of the anticancer drugs. Studies have, for instance, investigated the use of lipid-based nanoparticles to deliver docetaxel directly to HNC cells, such as clinical trial NCT02479178 [120]. Werner et al. [121] conjugated folate onto lipid polymer NP carriers of docetaxel, which enhanced their absorption in HNC cells in a time-dependent manner, making them effective ‘radiosensitizers’.

Studies by Rahman et al. [122] investigated the systemic delivery of small interfering RNA nanoparticles (siRNA NPs) targeting ribonucleotide reductase subunit M2 (RRM2), and assessed the effective dosing and prolonged knockdown of the targeted protein to achieve better therapeutic effect in HNSCC. siRNA NPs accumulated in HNSCC tumors and maintained gene knockdown for at least 10 days. This treatment significantly reduced tumor progression by inhibiting cell proliferation and inducing apoptosis in a mouse xenograft model [122]. The system that relies on siRNA NP-based therapy, consisting of siRNA encapsulated in a cyclodextrin-based polymer NP, was denoted CALAA-01, and was one of the first siRNA-based NP therapies that entered a phase I clinical trial (NCT00689065) to treat patients with solid cancers, though it was terminated due to safety concerns. Another study using a mouse model demonstrated that siRNA-based therapy using pH-responsive NPs can protect salivary glands (SGs) from radiation damage. Delivering Nkcc1 (targets Nkcc1 sodium–potassium–chloride cotransporter 1) and Pkcδ (protein kinase C delta) siRNAs to submandibular glands (SMGs) reduced apoptosis and improved saliva secretion for up to three months after radiation, suggesting that siRNA NPs could be an effective way to protect salivary glands from radiation damage [123]. More recently, NPs have been employed to deliver siRNA molecules that silence specific genes involved in HNC progression. This strategy has shown potential in inhibiting tumor growth in preclinical studies. Kampel et al. [124] demonstrated the therapeutic efficacy of anti-E6/E7 siRNA (E6, E7—oncogenes expressed in HPV-positive HNCs) delivered via targeted lipid-based nanoparticles (tLNPs) in an HPV-positive tumor model. The conjugation of anti-EGFR antibodies to the LNPs enhanced both the delivery of the siRNA and its anti-tumor effects, leading to a 50% greater reduction in tumor volume compared to control treatments (LNPs, which are coated with isotype control antibodies), with superior suppression of HPV oncogenes and increased apoptosis in both in vitro and in vivo models [124].

Recent studies focus also on gold NPs (AuNPs), which have the potential to enhance RT efficacy in HNCs by acting as ‘radiosensitizers’, allowing for a reduced total radiation dose and minimizing radiation-related toxicity [125]. The study by Huynh et al. [125] suggests that AuNP radiosensitization could improve RT outcomes in HNC patients by helping to overcome tumor hypoxia and the rapid regrowth of cancer cells, ultimately reducing the required radiation dose and minimizing toxicity. These findings highlight the potential of AuNPs to enhance the therapeutic ratio, particularly in RT given in smaller and more frequent doses, and support further investigation into clinical applications [125]. Other studies have also explored AuNPs as well as other metal-based NPs, including gadolinium, silver, and metal oxide-based NPs such as hafnium, zinc oxide, and iron oxide, in the context of radiosensitization for HNC treatment (recently reviewed in [126]). Recently, the use of the hafnium oxide-containing NP NBTXR3 was evaluated in a phase II clinical trial to evaluate the effect of NBTXR3 and radiation therapy in combination with pembrolizumab in patients with R/M-HNSCC (NCT04862455), holding promise in increasing RT efficacy. NBTXR3, activated by RT in combination with anti-PD-1 therapy, recently entered a phase I clinical trial, including a cohort of patients with locoregionally recurrent (LRR)- or R/M-HNSCC who are resistant to anti-PD-1 therapy, with injectable lesions located in the head and neck area, lung, or liver (NCT03589339).

Interestingly, in recent years the use of NPs in photothermal therapy (PTT), a minimally invasive cancer treatment, has also been studied for various cancers, including HNCs. The finding by Bu et al. [127] suggests that matrix metalloproteinase-degradable (MMP-degradable) gelatin nanoparticles (Gel-Ns) effectively release the encapsulated photosensitizer indocyanine green (ICG) and signal transducer and activator of transcription 3 (STAT3) inhibitor NSC74859 in tumor tissue, enabling a dual approach for HNSCC treatment, namely PTT and immunotherapy. Upon near-infrared (NIR) irradiation, the released ICG facilitates efficient tumor destruction, while NSC potentiates immunity, leading to enhanced therapeutic anticancer efficacy. In two HNSCC mouse models, Gel-N-ICG treatment significantly inhibited tumor growth without noticeable influence on the body weight of animals, highlighting the potential of protease-responsive nanoplatforms in overcoming the challenges of TME [127].

Additionally, current research is also exploring the use of mRNA nano-vaccines in preclinical models of HNC, either alone or in combination therapies. For instance, a recent study by Salomon et al. [128] used the novel ribonucleic acid lipoplex (RNA-LPX)-based HPV16 vaccine, E7 RNA-LPX, in combination with local radiotherapy (LRT), and demonstrated its enhanced anti-tumor efficacy in HPV16-positive cancer models. The combination therapy converts ‘cold’ tumors into immunologically ‘hot’ ones by increasing intratumoral CD8+ T cell infiltration, while LRT reduces tumor mass and intratumoral hypoxia, making tumor cells more susceptible to immune-mediated killing. Such vaccines are considered a promising therapeutic approach for HPV16-positive cancer patients, as highlighted by the study of Salomon et al. [128] and a previous study by Grunwitz et al. who also showed that HPV16 E7 RNA-LPX vaccine effectively primes HPV16-specific CD8+ T cells, leading to tumor remission in mice and enhanced tumor immunity [129].

### 3.4. PROTAC Technology

Apart from traditional targeted therapies, immunotherapy or the use of nanotechnology, targeted protein degradation (TPD) emerged as an innovative, promising therapeutic strategy to selectively degrade disease-related proteins. This approach relies primarily on the cell’s natural protein degradation mechanisms, such as the ubiquitin–proteasome system (UPS), to selectively remove proteins related to disease states, including cancer development (recently reviewed in [130,131,132]). The concept of TPD originated in 1999 via a patent application of Proteinex, Inc., Gaithersburg, MD (US) [133]. In 2001, the ‘degraders’ termed PROTACs were described by Raymond J. Deshaies and Craig M. Crews, with co-investigators [134], as chimeric molecules targeting the protein of interest (POI, in this case Met-AP-2—methionine aminopeptidase-2) to the ubiquitin ligase complex Skp1-Cullin-F box (SCF, containing Hrt1) for ubiquitylation and degradation, opening a new era of extensive research in the TPD field. Unlike traditional inhibitors that block protein function, PROTACs simultaneously bind to a target protein and an E3 ubiquitin ligase, enabling the elimination of POIs. This offers several advantages over traditional therapies, including the potential to target the so-called ‘undruggable’ proteins [135,136] and to overcome resistance mechanisms associated with conventional inhibitors [137,138].

**Figure 6:**
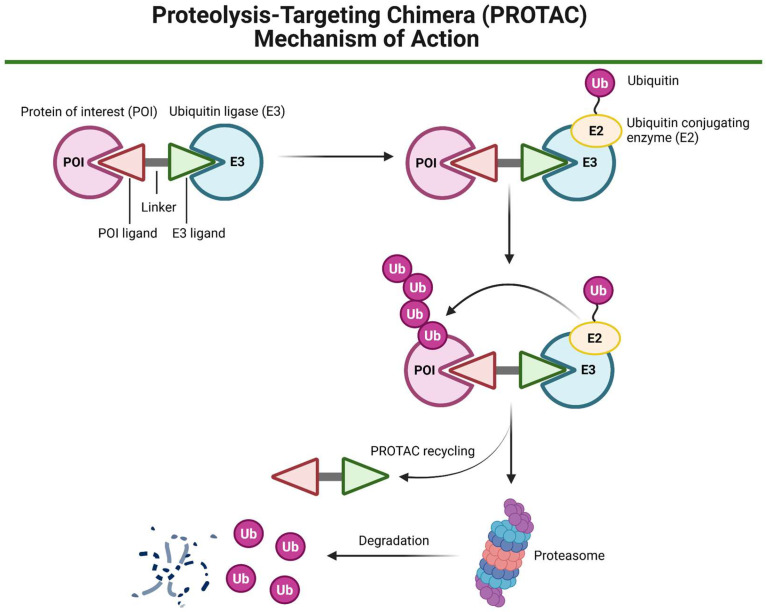
A schematic representation of the proteolysis-targeting chimera (PROTAC) mechanism of action. A PROTAC molecule consists of a ligand for ubiquitin ligase (E3) recruitment, a linker, and a second ligand that binds to the target protein of interest (POI). The PROTAC simultaneously engages both the POI and the E3 ligase, facilitating the formation of a ternary complex. Once this complex is formed, ubiquitin (Ub) molecules are transferred from the ubiquitin-conjugating enzyme (E2) to the POI in an E3-dependent manner, leading to polyubiquitination. The polyubiquitinated POI is then subjected to proteasomal degradation by the 26S proteasome. Created in BioRender. Blaszczak, E. (2025) (https://BioRender.com/).

Recently, PROTAC technology (Figure 6) has also been explored in treating HNC. The comparison of targeted therapies versus PROTAC technology in the context of HNC treatment is presented in Table 2. One notable example for HNSCC involves the development of a PROTAC, TSM-1, designed based on a natural small molecule toosendanin (TSN), to degrade STAT3, a protein often implicated in the progression of epithelial cancers, including HNSCC. TSM-1 has demonstrated robust anti-tumor effects in STAT3-dependent HNSCC models, particularly in patient-derived xenografts (PDXs) and patient-derived organoids (PDOs). Functional investigation has shown that TSM-1 promotes cell cycle arrest and apoptosis in tumor cells by reducing the expression of critical downstream STAT3 effectors [139]. This study demonstrates the successful proof-of-concept of the PROTAC strategy to degrade STAT3 in HNSCC, highlighting the potential of such degraders in cancer drug development.

HPV-related cancers, including cervical cancer and HNSCC, have the potential to metastasize to lymph nodes and in some cases also distant organs, e.g., the lungs. This may be driven by the E6 and E7 oncoproteins that promote metastasis by disrupting p53 and Rb tumor suppressors. Targeting E6 and E7 with PROTACs or proteolysis targeting antibodies (PROTABs) presents a promising strategy to inhibit metastasis initiation, potentially improving treatment outcomes in HPV-related cancers [140].

PROTAC technology has also been investigated in combination therapies. One such study developed a novel ‘radiosensitizer’, RPB7H, which combines a PROTAC molecule (BPA771) with hafnium dioxide NPs to enhance the effectiveness of RT in treating HNSCC. This approach targets the BRD4-RAD51AP1 pathway, making HNSCC cells more sensitive to RT by increasing DNA damage and preventing DNA repair. In animal models, RPB7H nanoparticles selectively accumulate in tumor tissues, and when combined with X-ray radiation, they significantly reduce tumor growth, offering a promising strategy to improve RT outcomes in HNSCC [141]. Although PROTAC compounds hold promise in precision and personalized medicine, they are not devoid of limitations. Translation into clinical practice may be limited due to cancer heterogeneity [142]. Owing to their structural properties, PROTACs also face limitations, such as low solubility and limited cellular permeability in vivo [143]. Understanding the full mechanism of action requires detailed investigation. Moreover, their efficacy and safety need to be assessed across diverse cancer types [142].

**Table 2:** The comparison of targeted therapies versus PROTAC technology in HNCs.

Feature	Traditional Inhibitors	Example References	PROTACs	Example References
Mechanism of action	Inhibition of the activity of oncogenic proteins	[32]	Induction of oncogenic proteins’ degradation	[131,144]
Examples of HNC targets	EGFR, PD-L1, MET, PI3K	[32]	STAT3, LZK (leucine zipper-bearing kinase)	[139,145]
Therapy effect duration	Dependent on pharmacokinetic exposure; continuous presence of the drug required	[146]	Longer effect (prolonged suppression)	[147]
Selectivity	Common off-target effects	[148]	Higher specificity due to the E3 ligase recruitment	[149]
HNC resistance	Frequent due to, e.g., EGFR mutations, MET pathway activation	[150,151]	Potentially lower due to protein degradation	[137]
Clinical stage in HNC	Some FDA-approved, e.g., cetuximab	[47]	Still limited for this cancer type	[142]

## 4. Brief Summary and Future Perspectives

HNCs pose significant treatment challenges mainly due to their high heterogeneity, relatively late diagnosis, resistance to conventional therapies, and high recurrence rate. The pathogenesis is even more complex because of signaling pathways’ abnormalities, TME inhibition, and genomic or epigenetic changes [82,152]. Moreover, risk factors, including infection with HPV, smoking, or alcohol abuse play a significant role in the development and progression of this complex disease [82]. In the light of the discussed limitations of conventional therapies, there is a constant need to develop novel approaches to reduce the problem of resistance and improve patient outcomes and quality of life. However, important questions remain: How can these new therapies be optimized in combination with standard treatments to enhance efficacy while minimizing these adverse effects? How can personalized medicine approaches, guided by molecular profiling and biomarker-driven strategies, with the possible aid of AI, transform patient care? Additionally, what novel molecular targets can be exploited to develop more effective and less toxic treatments for HNCs?

Targeted therapy using, e.g., EGFR inhibitors and immunotherapy with, e.g., ICIs, has been approved as a treatment for HNC; however, some patients are resistant to these therapies. Chemotherapy combined with immunotherapy has also shown the potential to modulate immune responses, and optimizing the sequence and timing of these treatments could significantly improve outcomes [153]. The search for molecular targets continues, with some being already tested in phase III clinical trials for HNCs, for instance, VEGFR, but with no significant survival benefits [73]. The integration of emerging technologies like NPs and PROTACs may overcome some of the limitations of conventional therapies. However, more studies are needed to assess their efficacy and safety for HNC treatment. The assistance of artificial intelligence (AI), particularly in intensity-modulated radiation therapy, may enhance RT precision, improving tumor targeting while minimizing damage to healthy tissues [154].

## 5. Conclusions

The diverse, yet aggressive nature of head and neck cancers has made it difficult to identify effective molecular targets for developing improved therapies that could significantly help many different patients. Resistance to conventional treatments, such as chemotherapy and radiotherapy, continues to be a major challenge, contributing to poor survival rates and severe toxicities that negatively impact patients’ quality of life. Understanding the molecular mechanisms underlying therapy resistance is thus crucial for advancing treatment strategies. Emerging therapies, including immune checkpoint inhibitors, PROTAC-based approaches, and novel targeted agents, offer new hope for overcoming resistance and improving patient outcomes. Addressing these challenges requires a multidisciplinary approach, integrating molecular oncology, clinical research, bioinformatic approaches, and personalized medicine to define therapeutic strategies and advance more effective treatment options for HNC patients.

## Figures and Tables

**Table 1:** The selected FDA-approved compounds and compounds in clinical trials for HNC treatment as targeted therapy or immunotherapy.

Compound	Class	Mechanism of Action	Exemplified Clinical Trial(s)	Use in HNC Therapy	FDA Approval	References
Cetuximab	Monoclonal antibody	Targets EGFR	NCT00004227	R/M-HNCs alone and in combination therapies	Approved	[47]
Cetuximab with cisplatin or carboplatin and 5-fluorouracil			NCT00122460			[48]
Pembrolizumab monotherapy or pembrolizumab, and cisplatin or carboplatin in combination with 5-fluorouracil (‘pembro combo’)	Monoclonal antibody	Inhibits PD-1, enhances immune response	NCT02358031	R/M-HNSCC	Approved	[17]
Nivolumab	Monoclonal antibody	Inhibits PD-1, enhances immune response	NCT02105636	R/M-HNCs	Approved	[15]
Durvalumab with cetuximab	Monoclonal antibody	Inhibits PD-L1 interaction with PD-1, enhances immune response	NCT03691714	Previously treated R/M-HNSCC	Phase II clinical trial	[49]
Atezolizumab	Monoclonal antibody	Inhibits PD-L1, enhances immune response	NCT04939480	Local HNSCC	Phase II clinical trial	[50]
Tislelizumab with gemcitabine and cisplatin	Monoclonal antibody	Inhibits PD-1, enhances immune response	NCT03924986	R/M nasopharyngeal cancer (NPC)	Phase III clinical trial	[51]
Cemiplimab with platinum-doublet chemotherapy, and cetuximab	Monoclonal antibody	Inhibits PD-1, enhances immune response	NCT04722523	Locoregionally advanced (LA) HNSCC	Phase I clinical trial	[52]
Lenvatinib with cetuximab	Multikinase inhibitor	Inhibits VEGFR, FGFR, PDGFR, RET, and CD117 *	NCT03524326	R/M-HNSCC	Phase I clinical trial	[53]

* VEGFR—vascular endothelial growth factor receptor, FGFR—fibroblast growth factor receptor, PDGFR—platelet-derived growth factor receptor, RET—rearranged during transfection tyrosine kinase, CD117—cluster of differentiation 117/receptor tyrosine kinase type III. Information retrieved from the Clinical Trials database (URL: https://clinicaltrials.gov, accessed on 4 March 2025) and referred publications.

## Abbreviations

ADC	Antibody–drug conjugate
AI	Artificial intelligence
AKT	Serine/threonine kinase
AuNP	Gold nanoparticle
BiTE	Bispecific T-cell engagers
BsAbs	Bispecific antibodies
BRD4	Bromodomain-containing protein 4
CAR-NK	Chimeric antigen receptor natural killer
CAR-T	Chimeric antigen receptor T
CD117	Cluster of differentiation 117
CDDP	Cisplatin
CRT	Chemoradiotherapy
CTLA-4	Cytotoxic T-lymphocyte-associated protein 4
DNMT	DNA methyltransferase
EBV	Epstein–Barr virus
EGFR	Epidermal growth factor receptor
EPR	Enhanced permeability and retention
ERK	Extracellular signal-regulated kinase
EZH2	Enhancer of zeste homolog 2
FAS	Fatty acid synthase
FDA	Food and Drug Administration
FGFR	Fibroblast growth factor receptor
5-FU	5-Fluorouracil
Gel-N	Gelatin nanoparticles
HDAC	Histone deacetylase
HER2	Human epidermal growth factor receptor 2
HNC	Head and neck cancer
HNSCC	Head and neck squamous cell carcinoma
HPV	Human papillomavirus
ICB	Immune checkpoint blockage
ICG	Indocyanine green
ICI	Immune checkpoint inhibitor
IMRT	Intensity-modulated radiotherapy
ITGB6	Integrin subunit beta 6
JAK	Janus kinase
LA	Locoregionally advanced
LRT	Local radiotherapy
MEK	Mitogen-activated protein kinase
MET	Mesenchymal–epithelial transition factor
Met-AP-2	Methionine aminopeptidase-2
MMP	Matrix metalloproteinase
mRNA	Messenger RNA
mTOR	Mechanistic target of rapamycin
NIR	Near-infrared
Nkcc1	Sodium–potassium–chloride cotransporter 1
NP	Nanoparticle
NPC	Nasopharyngeal cancer
OS	Overall survival
OSCC	Oral squamous cell carcinoma
PCC	Paclitaxel, carboplatin, and cetuximab
PD-1	Programmed cell death 1
PDGFR	Platelet-derived growth factor receptor
PD-L1	Programmed death-ligand 1
PDO	Patient-derived organoids
PDX	Patient-derived xenografts
PFS	Progression-free survival
PI3K	Phosphatidylinositol 3-kinase
Pkcδ	Protein kinase C delta
POI	Protein of interest
PROTAB	Proteolysis-targeting antibody
PROTAC	Proteolysis-targeting chimera
PTT	Photothermal therapy
R/M	Recurrent or metastatic
Raf	Rapidly accelerated fibrosarcoma
RET	Rearranged during transfection
RNA-LPX	Ribonucleic acid lipoplex
RR	Response rates
RRM2	Ribonucleotide reductase subunit M2
RT	Radiotherapy
SCF	Skp1-Cullin-F box
SG	Salivary glands
siRNA	Small interfering RNA
SMG	Submandibular glands
SQLE	Squalene epoxidase
STAT3	Signal transducer and activator of transcription 3
tLNPs	Targeted lipid-based nanoparticles
TAA	Tumor-associated antigens
TME	Tumor microenvironment
TPD	Targeted protein degradation
Trop-2	Trophoblast cell surface antigen 2
TSA	Tumor-specific antigens
TSN	Toosendanin
UPS	Ubiquitin-proteasome system
VEGF	Vascular endothelial growth factor
VEGFR	Vascular endothelial growth factor receptor

## References

[1] Mody M.D., Rocco J.W., Yom S.S., Haddad R.I., Saba N.F. (2021). Head and Neck Cancer. Lancet.

[2] Gormley M., Creaney G., Schache A., Ingarfield K., Conway D.I. (2022). Reviewing the Epidemiology of Head and Neck Cancer: Definitions, Trends and Risk Factors. Br. Dent. J..

[3] Wang Y., Han J., Zhu Y., Huang N., Qu N. (2025). New Advances in the Therapeutic Strategy of Head and Neck Squamous Cell Carcinoma: A Review of Latest Therapies and Cutting-Edge Research. Biochim. Biophys. Acta Rev. Cancer.

[4] Bray F., Laversanne M., Sung H., Ferlay J., Siegel R.L., Soerjomataram I., Jemal A. (2024). Global Cancer Statistics 2022: GLOBOCAN Estimates of Incidence and Mortality Worldwide for 36 Cancers in 185 Countries. CA Cancer J. Clin..

[5] Ferlay J., Colombet M., Soerjomataram I., Mathers C., Parkin D.M., Piñeros M., Znaor A., Bray F. (2019). Estimating the Global Cancer Incidence and Mortality in 2018: GLOBOCAN Sources and Methods. Int. J. Cancer.

[6] Bravi F., Lee Y.-C.A., Hashibe M., Boffetta P., Conway D.I., Ferraroni M., La Vecchia C., Edefonti V., INHANCE Consortium investigators (2021). Lessons Learned from the INHANCE Consortium: An Overview of Recent Results on Head and Neck Cancer. Oral Dis..

[7] Gillison M.L., Chaturvedi A.K., Anderson W.F., Fakhry C. (2015). Epidemiology of Human Papillomavirus-Positive Head and Neck Squamous Cell Carcinoma. J. Clin. Oncol..

[8] Yan F., Knochelmann H.M., Morgan P.F., Kaczmar J.M., Neskey D.M., Graboyes E.M., Nguyen S.A., Ogretmen B., Sharma A.K., Day T.A. (2020). The Evolution of Care of Cancers of the Head and Neck Region: State of the Science in 2020. Cancers.

[9] Menezes F.D.S., Fernandes G.A., Antunes J.L.F., Villa L.L., Toporcov T.N. (2021). Global Incidence Trends in Head and Neck Cancer for HPV-Related and -Unrelated Subsites: A Systematic Review of Population-Based Studies. Oral Oncol..

[10] Ferris R.L., Saba N.F., Gitlitz B.J., Haddad R., Sukari A., Neupane P., Morris J.C., Misiukiewicz K., Bauman J.E., Fenton M. (2018). Effect of Adding Motolimod to Standard Combination Chemotherapy and Cetuximab Treatment of Patients With Squamous Cell Carcinoma of the Head and Neck: The Active8 Randomized Clinical Trial. JAMA Oncol..

[11] Elrefaey S., Massaro M.A., Chiocca S., Chiesa F., Ansarin M. (2014). HPV in Oropharyngeal Cancer: The Basics to Know in Clinical Practice. Acta Otorhinolaryngol. Ital..

[12] Hu H., Li B., Wang J., Tan Y., Xu M., Xu W., Lu H. (2023). New Advances into Cisplatin Resistance in Head and Neck Squamous Carcinoma: Mechanisms and Therapeutic Aspects. Biomed. Pharmacother..

[13] Cooper J.S., Zhang Q., Pajak T.F., Forastiere A.A., Jacobs J., Saxman S.B., Kish J.A., Kim H.E., Cmelak A.J., Rotman M. (2012). Long-Term Follow-up of the RTOG 9501/intergroup Phase III Trial: Postoperative Concurrent Radiation Therapy and Chemotherapy in High-Risk Squamous Cell Carcinoma of the Head and Neck. Int. J. Radiat. Oncol. Biol. Phys..

[14] Brook I. (2020). Late Side Effects of Radiation Treatment for Head and Neck Cancer. Radiat. Oncol. J..

[15] Ferris R.L., Blumenschein G., Fayette J., Guigay J., Colevas A.D., Licitra L., Harrington K., Kasper S., Vokes E.E., Even C. (2016). Nivolumab for Recurrent Squamous-Cell Carcinoma of the Head and Neck. N. Engl. J. Med..

[16] Seiwert T.Y., Burtness B., Mehra R., Weiss J., Berger R., Eder J.P., Heath K., McClanahan T., Lunceford J., Gause C. (2016). Safety and Clinical Activity of Pembrolizumab for Treatment of Recurrent or Metastatic Squamous Cell Carcinoma of the Head and Neck (KEYNOTE-012): An Open-Label, Multicentre, Phase 1b Trial. Lancet Oncol..

[17] Burtness B., Harrington K.J., Greil R., Soulières D., Tahara M., de Castro G., Psyrri A., Basté N., Neupane P., Bratland Å. (2019). Pembrolizumab Alone or with Chemotherapy versus Cetuximab with Chemotherapy for Recurrent or Metastatic Squamous Cell Carcinoma of the Head and Neck (KEYNOTE-048): A Randomised, Open-Label, Phase 3 Study. Lancet.

[18] Johnson D.B., Nebhan C.A., Moslehi J.J., Balko J.M. (2022). Immune-Checkpoint Inhibitors: Long-Term Implications of Toxicity. Nat. Rev. Clin. Oncol..

[19] Chow L.Q.M. (2020). Head and Neck Cancer. N. Engl. J. Med..

[20] Kanno Y., Chen C.-Y., Lee H.-L., Chiou J.-F., Chen Y.-J. (2021). Molecular Mechanisms of Chemotherapy Resistance in Head and Neck Cancers. Front. Oncol..

[21] Tan Y., Wang Z., Xu M., Li B., Huang Z., Qin S., Nice E.C., Tang J., Huang C. (2023). Oral Squamous Cell Carcinomas: State of the Field and Emerging Directions. Int. J. Oral Sci..

[22] Runnels J., Bloom J.R., Hsieh K., Dickstein D.R., Shi Y., Jones B.M., Lehrer E.J., Bakst R.L. (2023). Combining Radiotherapy and Immunotherapy in Head and Neck Cancer. Biomedicines.

[23] McMahon J., Handley T.P.B., Bobinskas A., Elsapagh M., Anwar H.S., Ricciardo P.V., McLaren A., Davis R., Syyed N., MacIver C. (2017). Postoperative Complications after Head and Neck Operations That Require Free Tissue Transfer—Prevalent, Morbid, and Costly. Br. J. Oral Maxillofac. Surg..

[24] Nieminen T., Tapiovaara L., Bäck L., Lindford A., Lassus P., Lehtonen L., Mäkitie A., Keski-Säntti H. (2024). Enhanced Recovery after Surgery (ERAS) Protocol Improves Patient Outcomes in Free Flap Surgery for Head and Neck Cancer. Eur. Arch. Otorhinolaryngol..

[25] Alterio D., Marvaso G., Ferrari A., Volpe S., Orecchia R., Jereczek-Fossa B.A. (2019). Modern Radiotherapy for Head and Neck Cancer. Semin. Oncol..

[26] Budach V., Thieme A. (2023). Proton Therapy for Head and Neck Cancer. Critical Issues in Head and Neck Oncology.

[27] Kiafi P., Chalkia M., Kouri M.A., Patatoukas G., Kollaros N., Kougioumtzopoulou A., Nikolatou-Galitis O., Kyrodimos E., Perisanidis C., Kouloulias V. (2024). Photon vs. Proton Radiation Therapy in Head and Neck Cancer: A Review of Dosimetric Advantages and Patient Quality of Life. J. Cancer Metastasis Treat..

[28] Iorio G.C., Denaro N., Livi L., Desideri I., Nardone V., Ricardi U. (2024). Editorial: Advances in Radiotherapy for Head and Neck Cancer. Front. Oncol..

[29] Jumaniyazova E., Smyk D., Vishnyakova P., Fatkhudinov T., Gordon K. (2022). Photon- and Proton-Mediated Biological Effects: What Has Been Learned?. Life.

[30] Meyer F., Fortin A., Wang C.S., Liu G., Bairati I. (2012). Predictors of Severe Acute and Late Toxicities in Patients with Localized Head-and-Neck Cancer Treated with Radiation Therapy. Int. J. Radiat. Oncol. Biol. Phys..

[31] Minicucci E.M., da Silva G.N., Salvadori D.M.F. (2014). Relationship between Head and Neck Cancer Therapy and Some Genetic Endpoints. World J. Clin. Oncol..

[32] Li Q., Tie Y., Alu A., Ma X., Shi H. (2023). Targeted Therapy for Head and Neck Cancer: Signaling Pathways and Clinical Studies. Signal Transduct. Target. Ther..

[33] Brockstein B.E., Vokes E.E. (1999). Oral Chemotherapy in Head and Neck Cancer. Drugs.

[34] Guidi A., Codecà C., Ferrari D. (2018). Chemotherapy and Immunotherapy for Recurrent and Metastatic Head and Neck Cancer: A Systematic Review. Med. Oncol..

[35] Forastiere A.A., Metch B., Schuller D.E., Ensley J.F., Hutchins L.F., Triozzi P., Kish J.A., McClure S., VonFeldt E., Williamson S.K. (1992). Randomized Comparison of Cisplatin plus Fluorouracil and Carboplatin plus Fluorouracil versus Methotrexate in Advanced Squamous-Cell Carcinoma of the Head and Neck: A Southwest Oncology Group Study. J. Clin. Oncol..

[36] Jacobs C., Lyman G., Velez-García E., Sridhar K.S., Knight W., Hochster H., Goodnough L.T., Mortimer J.E., Einhorn L.H., Schacter L. (1992). A Phase III Randomized Study Comparing Cisplatin and Fluorouracil as Single Agents and in Combination for Advanced Squamous Cell Carcinoma of the Head and Neck. J. Clin. Oncol..

[37] Ma S.J., Zhu S., Virk J., Koempel A., Bhateja P., Gogineni E., Baliga S., Konieczkowski D., Mitchell D., Jhawar S. (2024). Weekly Cisplatin Cycles and Outcomes for Chemoradiation in Head and Neck Cancer. JAMA Netw. Open.

[38] Catimel G., Verweij J., Mattijssen V., Hanauske A., Piccart M., Wanders J., Franklin H., Le Bail N., Clavel M., Kaye S.B. (1994). Docetaxel (Taxotere): An Active Drug for the Treatment of Patients with Advanced Squamous Cell Carcinoma of the Head and Neck. EORTC Early Clinical Trials Group. Ann. Oncol..

[39] Dreyfuss A.I., Clark J.R., Norris C.M., Rossi R.M., Lucarini J.W., Busse P.M., Poulin M.D., Thornhill L., Costello R., Posner M.R. (1996). Docetaxel: An Active Drug for Squamous Cell Carcinoma of the Head and Neck. J. Clin. Oncol..

[40] Dasari S., Tchounwou P.B. (2014). Cisplatin in Cancer Therapy: Molecular Mechanisms of Action. Eur. J. Pharmacol..

[41] Kitamura N., Sento S., Yoshizawa Y., Sasabe E., Kudo Y., Yamamoto T. (2020). Current Trends and Future Prospects of Molecular Targeted Therapy in Head and Neck Squamous Cell Carcinoma. Int. J. Mol. Sci..

[42] Forman R., Deshpande H., Burtness B., Bhatia A.K. (2022). Efficacy and Toxicity of Weekly Paclitaxel, Carboplatin, and Cetuximab as Induction Chemotherapy or in Cases of Metastases or Relapse for Head and Neck Cancer with a Focus on Elderly or Frail Patients. Head Neck.

[43] Lam L., Samman N. (2013). Speech and Swallowing Following Tongue Cancer Surgery and Free Flap Reconstruction—A Systematic Review. Oral Oncol..

[44] Sharma S., Kumar Upadhyay A., Prakash A., Singodia P., Ravi Kiran S., Shankar R. (2024). Treatment Complications of Head and Neck Cancers and Rehabilitation Measures: A Narrative Review. Cureus.

[45] Brook I. (2021). Early Side Effects of Radiation Treatment for Head and Neck Cancer. Cancer Radiother..

[46] Nguyen N.P., Sallah S., Karlsson U., Antoine J.E. (2002). Combined Chemotherapy and Radiation Therapy for Head and Neck Malignancies: Quality of Life Issues. Cancer.

[47] Bonner J.A., Harari P.M., Giralt J., Azarnia N., Shin D.M., Cohen R.B., Jones C.U., Sur R., Raben D., Jassem J. (2006). Radiotherapy plus Cetuximab for Squamous-Cell Carcinoma of the Head and Neck. N. Engl. J. Med..

[48] Vermorken J.B., Mesia R., Rivera F., Remenar E., Kawecki A., Rottey S., Erfan J., Zabolotnyy D., Kienzer H.-R., Cupissol D. (2008). Platinum-Based Chemotherapy plus Cetuximab in Head and Neck Cancer. N. Engl. J. Med..

[49] Gulati S., Crist M., Riaz M.K., Takiar V., Lehn M., Monroe I., Palackdharry S., Kurtzweil N., Jandarov R., Harun N. (2023). Durvalumab plus Cetuximab in Patients with Recurrent or Metastatic Head and Neck Squamous Cell Carcinoma: An Open-Label, Nonrandomized, Phase II Clinical Trial. Clin. Cancer Res..

[50] Gong Y., Bao L., Xu T., Yi X., Chen J., Wang S., Pan Z., Huang P., Ge M. (2023). The Tumor Ecosystem in Head and Neck Squamous Cell Carcinoma and Advances in Ecotherapy. Mol. Cancer.

[51] Yang Y., Pan J., Wang H., Zhao Y., Qu S., Chen N., Chen X., Sun Y., He X., Hu C. (2023). Tislelizumab plus Chemotherapy as First-Line Treatment for Recurrent or Metastatic Nasopharyngeal Cancer: A Multicenter Phase 3 Trial (RATIONALE-309). Cancer Cell.

[52] Wong W., Cracchiolo J.R., Riaz N., Ganly I., Sherman E.J., Ho A.L., Morris L., Ghossein R.A., Haque S., Hung K.W. (2023). Neoadjuvant Cemiplimab with Platinum-Doublet Chemotherapy and Cetuximab to de-Escalate Surgery and Omit Adjuvant Radiation in Locoregionally Advanced Head & Neck Squamous Cell Carcinoma (HNSCC). J. Clin. Oncol..

[53] Dunn L., Ho A.L., Eng J., Michel L.S., Fetten J.V., Warner E., Kriplani A., Zhi W.I., Ng K.K., Haque S. (2020). A Phase I/Ib Study of Lenvatinib and Cetuximab in Patients with Recurrent/metastatic (R/M) Head and Neck Squamous Cell Carcinoma (HNSCC). J. Clin. Oncol..

[54] Hendler F.J., Ozanne B.W. (1984). Human Squamous Cell Lung Cancers Express Increased Epidermal Growth Factor Receptors. J. Clin. Investig..

[55] Zimmermann M., Zouhair A., Azria D., Ozsahin M. (2006). The Epidermal Growth Factor Receptor (EGFR) in Head and Neck Cancer: Its Role and Treatment Implications. Radiat. Oncol..

[56] Robert F., Ezekiel M.P., Spencer S.A., Meredith R.F., Bonner J.A., Khazaeli M.B., Saleh M.N., Carey D., LoBuglio A.F., Wheeler R.H. (2001). Phase I Study of Anti--Epidermal Growth Factor Receptor Antibody Cetuximab in Combination with Radiation Therapy in Patients with Advanced Head and Neck Cancer. J. Clin. Oncol..

[57] Baselga J., Pfister D., Cooper M.R., Cohen R., Burtness B., Bos M., D’Andrea G., Seidman A., Norton L., Gunnett K. (2000). Phase I Studies of Anti-Epidermal Growth Factor Receptor Chimeric Antibody C225 Alone and in Combination with Cisplatin. J. Clin. Oncol..

[58] Lefebvre J.L., Pointreau Y., Rolland F., Alfonsi M., Baudoux A., Sire C., de Raucourt D., Malard O., Degardin M., Tuchais C. (2013). Induction Chemotherapy Followed by Either Chemoradiotherapy or Bioradiotherapy for Larynx Preservation: The TREMPLIN Randomized Phase II Study. J. Clin. Oncol..

[59] Ghi M.G., Paccagnella A., Ferrari D., Foa P., Alterio D., Codecà C., Nolè F., Verri E., Orecchia R., Morelli F. (2017). Induction TPF Followed by Concomitant Treatment versus Concomitant Treatment Alone in Locally Advanced Head and Neck Cancer. A Phase II-III Trial. Ann. Oncol..

[60] Nakano K. (2021). Progress of Molecular Targeted Therapy for Head and Neck Cancer in Clinical Aspects. Mol. Biomed..

[61] Grade H., Grade H. (2010). StatBite: Radiation Therapy plus Cetuximab: Skin Reactions from 71 Head and Neck Cancer Patients from 11 Institutions in Europe. J. Natl. Cancer Inst..

[62] Specenier P., Vermorken J.B. (2013). Cetuximab: Its Unique Place in Head and Neck Cancer Treatment. Biologics.

[63] Waris W., Naik S., Idrees I., Taha H., Camosino L., Mehrishi A., Saif M.W. (2009). Severe Cutaneous Reaction to Cetuximab with Possible Association with the Use of over-the-Counter Skin Care Products in a Patient with Oropharyngeal Cancer. Cutan. Ocul. Toxicol..

[64] Hansel T.T., Kropshofer H., Singer T., Mitchell J.A., George A.J.T. (2010). The Safety and Side Effects of Monoclonal Antibodies. Nat. Rev. Drug Discov..

[65] Kalanjeri S., Stover D., Lee R. (2012). Acute Eosinophilic Pneumonia Associated with Cetuximab. Chest.

[66] Wang L., Chen Y.-Z., Shi D., Shi X.-Y., Zou Z., Zhao J.-H. (2011). Incidence and Risk of Severe Neutropenia in Advanced Cancer Patients Treated with Cetuximab: A Meta-Analysis. Drugs R D.

[67] Cui R., Chu L., Liu Z.-Q., Xiao Y.-Y., Zhu X.-L., Chen Y.-J., Xu Q. (2016). Hematologic Toxicity Assessment in Solid Tumor Patients Treated with Cetuximab: A Pooled Analysis of 18 Randomized Controlled Trials. Int. J. Cancer.

[68] Stanbouly D., Philipone E., Morlandt A.B., Kaleem A., Chuang S.-K., Patel N. (2022). Adverse Events Secondary to Cetuximab Therapy in Head & Neck Cancer Therapy and Risk Factors for Serious Outcomes. Oral Oncol..

[69] Singh P., Contente M., Bennett B., Hall J., Bailey H., Bailey A., Zarrelli L., Polanco Sanchez C. (2021). Real-World Treatment Patterns and Outcomes in Patients with Head and Neck Cancer: Point-in-Time Survey of Oncologists in Italy and Spain. Adv. Ther..

[70] Ng K., Metcalf R., Sacco J., Kong A., Wheeler G., Forsyth S., Bhat R., Ward J., Ensell L., Lowe H. (2023). Protocol for the EACH Trial: A Multicentre Phase II Study Evaluating the Safety and Antitumour Activity of the Combination of Avelumab, an Anti-PD-L1 Agent, and Cetuximab, as Any Line Treatment for Patients with Recurrent/metastatic Head and Neck Squamous Cell Cancer (HNSCC) in the UK. BMJ Open.

[71] Chung C.H., Li J., Steuer C.E., Bhateja P., Johnson M., Masannat J., Poole M.I., Song F., Hernandez-Prera J.C., Molina H. (2022). Phase II Multi-Institutional Clinical Trial Result of Concurrent Cetuximab and Nivolumab in Recurrent And/or Metastatic Head and Neck Squamous Cell Carcinoma. Clin. Cancer Res..

[72] Dhillon S. (2020). Capmatinib: First Approval. Drugs.

[73] Argiris A., Li S., Savvides P., Ohr J.P., Gilbert J., Levine M.A., Chakravarti A., Haigentz M., Saba N.F., Ikpeazu C.V. (2019). Phase III Randomized Trial of Chemotherapy with or without Bevacizumab in Patients with Recurrent or Metastatic Head and Neck Cancer. J. Clin. Oncol..

[74] Xu X., Chen J., Li Y., Yang X., Wang Q., Wen Y., Yan M., Zhang J., Xu Q., Wei Y. (2021). Targeting Epigenetic Modulation of Cholesterol Synthesis as a Therapeutic Strategy for Head and Neck Squamous Cell Carcinoma. Cell Death Dis..

[75] Amiri M., Yousefnia S., Seyed Forootan F., Peymani M., Ghaedi K., Nasr Esfahani M.H. (2018). Diverse Roles of Fatty Acid Binding Proteins (FABPs) in Development and Pathogenesis of Cancers. Gene.

[76] Fang L.-Y., Wong T.-Y., Chiang W.-F., Chen Y.-L. (2010). Fatty-Acid-Binding Protein 5 Promotes Cell Proliferation and Invasion in Oral Squamous Cell Carcinoma: FABP5 Promotes Oral Cancer Cell Proliferation and Invasion. J. Oral Pathol. Med..

[77] Vidotto A., Polachini G.M., de Paula-Silva M., Oliani S.M., Henrique T., López R.V.M., Cury P.M., Nunes F.D., Góis-Filho J.F., de Carvalho M.B. (2018). Differentially Expressed Proteins in Positive versus Negative HNSCC Lymph Nodes. BMC Med. Genom..

[78] Agostini M., Silva S.D., Zecchin K.G., Coletta R.D., Jorge J., Loda M., Graner E. (2004). Fatty Acid Synthase Is Required for the Proliferation of Human Oral Squamous Carcinoma Cells. Oral Oncol..

[79] Agostini M., Almeida L.Y., Bastos D.C., Ortega R.M., Moreira F.S., Seguin F., Zecchin K.G., Raposo H.F., Oliveira H.C.F., Amoêdo N.D. (2014). The Fatty Acid Synthase Inhibitor Orlistat Reduces the Growth and Metastasis of Orthotopic Tongue Oral Squamous Cell Carcinomas. Mol. Cancer Ther..

[80] Perri F., Della Vittoria Scarpati G., Pontone M., Marciano M.L., Ottaiano A., Cascella M., Sabbatino F., Guida A., Santorsola M., Maiolino P. (2022). Cancer Cell Metabolism Reprogramming and Its Potential Implications on Therapy in Squamous Cell Carcinoma of the Head and Neck: A Review. Cancers.

[81] Ferris R.L. (2015). Immunology and Immunotherapy of Head and Neck Cancer. J. Clin. Oncol..

[82] Liu Y., Zhang N., Wen Y., Wen J. (2024). Head and Neck Cancer: Pathogenesis and Targeted Therapy. MedComm.

[83] Ferris R.L., Licitra L., Fayette J., Even C., Blumenschein G., Harrington K.J., Guigay J., Vokes E.E., Saba N.F., Haddad R. (2019). Nivolumab in Patients with Recurrent or Metastatic Squamous Cell Carcinoma of the Head and Neck: Efficacy and Safety in CheckMate 141 by Prior Cetuximab Use. Clin. Cancer Res..

[84] Friedman C.F., Proverbs-Singh T.A., Postow M.A. (2016). Treatment of the Immune-Related Adverse Effects of Immune Checkpoint Inhibitors: A Review. JAMA Oncol..

[85] Postow M.A., Sidlow R., Hellmann M.D. (2018). Immune-Related Adverse Events Associated with Immune Checkpoint Blockade. N. Engl. J. Med..

[86] Shi Y., Guo W., Wang W., Wu Y., Fang M., Huang X., Han P., Zhang Q., Dong P., Zhou X. (2024). Finotonlimab with Chemotherapy in Recurrent or Metastatic Head and Neck Cancer: A Randomized Phase 3 Trial. Nat. Med..

[87] Maron D.J., Fazio S., Linton M.F. (2000). Current Perspectives on Statins. Circulation.

[88] Seol S., Choi J.R., Choi B., Kim S., Jeon J.Y., Park K.N., Park J.H., Park M.W., Eun Y.-G., Park J.J. (2023). Effect of Statin Use on Head and Neck Cancer Prognosis in a Multicenter Study Using a Common Data Model. Sci. Rep..

[89] Demierre M.-F., Higgins P.D.R., Gruber S.B., Hawk E., Lippman S.M. (2005). Statins and Cancer Prevention. Nat. Rev. Cancer.

[90] Sheridan A., Wheeler-Jones C.P.D., Gage M.C. (2022). The Immunomodulatory Effects of Statins on Macrophages. Immuno.

[91] Kansal V., Burnham A.J., Kinney B.L.C., Saba N.F., Paulos C., Lesinski G.B., Buchwald Z.S., Schmitt N.C. (2023). Statin Drugs Enhance Responses to Immune Checkpoint Blockade in Head and Neck Cancer Models. J. Immunother. Cancer.

[92] Goossens P., Rodriguez-Vita J., Etzerodt A., Masse M., Rastoin O., Gouirand V., Ulas T., Papantonopoulou O., Van Eck M., Auphan-Anezin N. (2019). Membrane Cholesterol Efflux Drives Tumor-Associated Macrophage Reprogramming and Tumor Progression. Cell Metab..

[93] Vitale I., Manic G., Coussens L.M., Kroemer G., Galluzzi L. (2019). Macrophages and Metabolism in the Tumor Microenvironment. Cell Metab..

[94] Qiao X., Hu Z., Xiong F., Yang Y., Peng C., Wang D., Li X. (2023). Lipid Metabolism Reprogramming in Tumor-Associated Macrophages and Implications for Therapy. Lipids Health Dis..

[95] Zhang C., Li K., Zhu H., Cheng M., Chen S., Ling R., Wang C., Chen D. (2024). ITGB6 Modulates Resistance to Anti-CD276 Therapy in Head and Neck Cancer by Promoting PF4+ Macrophage Infiltration. Nat. Commun..

[96] Hu C., Liu M., Li Y., Zhao Y., Sharma A., Liu H., Schmidt-Wolf I.G.H. (2023). Recent Advances and Future Perspectives of CAR-T Cell Therapy in Head and Neck Cancer. Front. Immunol..

[97] Wang H.-Q., Fu R., Man Q.-W., Yang G., Liu B., Bu L.-L. (2023). Advances in CAR-T Cell Therapy in Head and Neck Squamous Cell Carcinoma. J. Clin. Med..

[98] Manzar G.S., Rafei H., Kumar B., Shanley M., Acharya S., Liu B., Xu A., Wang X.A., Islam S., Kaplan M. (2023). Radiation Therapy Sensitizes Head-and-Neck Cancer Cells to Killing by Chimeric Antigen Receptor (CAR)-NK Cells Targeting CD70. Int. J. Radiat. Oncol. Biol. Phys..

[99] Nowak J., Bentele M., Kutle I., Zimmermann K., Lühmann J.L., Steinemann D., Kloess S., Koehl U., Roßberg W., Ahmed A. (2023). CAR-NK Cells Targeting HER1 (EGFR) Show Efficient Anti-Tumor Activity against Head and Neck Squamous Cell Carcinoma (HNSCC). Cancers.

[100] Ciulean I.S., Fischer J., Quaiser A., Bach C., Abken H., Tretbar U.S., Fricke S., Koehl U., Schmiedel D., Grunwald T. (2023). CD44v6 Specific CAR-NK Cells for Targeted Immunotherapy of Head and Neck Squamous Cell Carcinoma. Front. Immunol..

[101] Jiménez-Labaig P., Rullan A., Hernando-Calvo A., Llop S., Bhide S., O’Leary B., Braña I., Harrington K.J. (2024). A Systematic Review of Antibody-Drug Conjugates and Bispecific Antibodies in Head and Neck Squamous Cell Carcinoma and Nasopharyngeal Carcinoma: Charting the Course of Future Therapies. Cancer Treat. Rev..

[102] Dewaele L., Fernandes R.A. (2025). Bispecific T-Cell Engagers for the Recruitment of T Cells in Solid Tumors: A Literature Review. Immunother. Adv..

[103] Vallera D.A., Felices M., McElmurry R., McCullar V., Zhou X., Schmohl J.U., Zhang B., Lenvik A.J., Panoskaltsis-Mortari A., Verneris M.R. (2016). IL15 Trispecific Killer Engagers (TriKE) Make Natural Killer Cells Specific to CD33+ Targets While Also Inducing Persistence, in Vivo Expansion, and Enhanced Function. Clin. Cancer Res..

[104] Gauthier L., Morel A., Anceriz N., Rossi B., Blanchard-Alvarez A., Grondin G., Trichard S., Cesari C., Sapet M., Bosco F. (2019). Multifunctional Natural Killer Cell Engagers Targeting NKp46 Trigger Protective Tumor Immunity. Cell.

[105] Gauthier L., Virone-Oddos A., Beninga J., Rossi B., Nicolazzi C., Amara C., Blanchard-Alvarez A., Gourdin N., Courta J., Basset A. (2023). Control of Acute Myeloid Leukemia by a Trifunctional NKp46-CD16a-NK Cell Engager Targeting CD123. Nat. Biotechnol..

[106] Demaria O., Gauthier L., Vetizou M., Blanchard Alvarez A., Vagne C., Habif G., Batista L., Baron W., Belaïd N., Girard-Madoux M. (2022). Antitumor Immunity Induced by Antibody-Based Natural Killer Cell Engager Therapeutics Armed with Not-Alpha IL-2 Variant. Cell Rep. Med..

[107] Stevanović S., Helman S.R., Wunderlich J.R., Langhan M.M., Doran S.L., Kwong M.L.M., Somerville R.P.T., Klebanoff C.A., Kammula U.S., Sherry R.M. (2019). A Phase II Study of Tumor-Infiltrating Lymphocyte Therapy for Human Papillomavirus-Associated Epithelial Cancers. Clin. Cancer Res..

[108] Jimeno A., Papa S., Haigentz M., Rodríguez-Moreno J., Schardt J., Fardis M., Finckenstein F.G., Fiaz R., Chen G., Cacovean A. (2020). 353 Safety and Efficacy of Tumor Infiltrating Lymphocytes (TIL, LN-145) in Combination with Pembrolizumab for Advanced, Recurrent or Metastatic HNSCC. J. Immunother. Cancer.

[109] O’Malley D., Lee S., Psyrri A., Sukari A., Thomas S., Wenham R., Gogas H., Jazaeri A., Monk B., Rose P. (2021). 492 Phase 2 Efficacy and Safety of Autologous Tumor-Infiltrating Lymphocyte (TIL) Cell Therapy in Combination with Pembrolizumab in Immune Checkpoint Inhibitor-Naïve Patients with Advanced Cancers. J. Immunother. Cancer.

[110] Albarrán V., San Román M., Pozas J., Chamorro J., Rosero D.I., Guerrero P., Calvo J.C., González C., García de Quevedo C., Pérez de Aguado P. (2024). Adoptive T Cell Therapy for Solid Tumors: Current Landscape and Future Challenges. Front. Immunol..

[111] Wang H., Yu J., Lu X., He X. (2016). Nanoparticle Systems Reduce Systemic Toxicity in Cancer Treatment. Nanomedicine.

[112] Ruiz-Pulido G., Medina D.I., Barani M., Rahdar A., Sargazi G., Baino F., Pandey S. (2021). Nanomaterials for the Diagnosis and Treatment of Head and Neck Cancers: A Review. Materials.

[113] Li J., Wang Q., Xia G., Adilijiang N., Li Y., Hou Z., Fan Z., Li J. (2023). Recent Advances in Targeted Drug Delivery Strategy for Enhancing Oncotherapy. Pharmaceutics.

[114] Bazak R., Houri M., Achy S.E., Hussein W., Refaat T. (2014). Passive Targeting of Nanoparticles to Cancer: A Comprehensive Review of the Literature. Mol. Clin. Oncol..

[115] Maeda H. (2015). Toward a Full Understanding of the EPR Effect in Primary and Metastatic Tumors as Well as Issues Related to Its Heterogeneity. Adv. Drug Deliv. Rev..

[116] Attia M.F., Anton N., Wallyn J., Omran Z., Vandamme T.F. (2019). An Overview of Active and Passive Targeting Strategies to Improve the Nanocarriers Efficiency to Tumour Sites. J. Pharm. Pharmacol..

[117] Bertrand N., Wu J., Xu X., Kamaly N., Farokhzad O.C. (2014). Cancer Nanotechnology: The Impact of Passive and Active Targeting in the Era of Modern Cancer Biology. Adv. Drug Deliv. Rev..

[118] Puri S., Mazza M., Roy G., England R.M., Zhou L., Nourian S., Anand Subramony J. (2023). Evolution of Nanomedicine Formulations for Targeted Delivery and Controlled Release. Adv. Drug Deliv. Rev..

[119] Li H.-X., Gong Y.-W., Yan P.-J., Xu Y., Qin G., Wen W.-P., Teng F.-Y. (2024). Revolutionizing Head and Neck Squamous Cell Carcinoma Treatment with Nanomedicine in the Era of Immunotherapy. Front. Immunol..

[120] Jurczyk M., Kasperczyk J., Wrześniok D., Beberok A., Jelonek K. (2022). Nanoparticles Loaded with Docetaxel and Resveratrol as an Advanced Tool for Cancer Therapy. Biomedicines.

[121] Werner M.E., Copp J.A., Karve S., Cummings N.D., Sukumar R., Li C., Napier M.E., Chen R.C., Cox A.D., Wang A.Z. (2011). Folate-Targeted Polymeric Nanoparticle Formulation of Docetaxel Is an Effective Molecularly Targeted Radiosensitizer with Efficacy Dependent on the Timing of Radiotherapy. ACS Nano.

[122] Rahman M.A., Amin A.R.M.R., Wang X., Zuckerman J.E., Choi C.H.J., Zhou B., Wang D., Nannapaneni S., Koenig L., Chen Z. (2012). Systemic Delivery of siRNA Nanoparticles Targeting RRM2 Suppresses Head and Neck Tumor Growth. J. Control. Release.

[123] Arany S., Benoit D.S.W., Dewhurst S., Ovitt C.E. (2013). Nanoparticle-Mediated Gene Silencing Confers Radioprotection to Salivary Glands in Vivo. Mol. Ther..

[124] Kampel L., Goldsmith M., Ramishetti S., Veiga N., Rosenblum D., Gutkin A., Chatterjee S., Penn M., Lerman G., Peer D. (2021). Therapeutic Inhibitory RNA in Head and Neck Cancer via Functional Targeted Lipid Nanoparticles. J. Control. Release.

[125] Huynh M., Kempson I., Bezak E., Phillips W. (2021). Predictive Modeling of Hypoxic Head and Neck Cancers during Fractionated Radiotherapy with Gold Nanoparticle Radiosensitization. Med. Phys..

[126] Dubey P., Sertorio M., Takiar V. (2022). Therapeutic Advancements in Metal and Metal Oxide Nanoparticle-Based Radiosensitization for Head and Neck Cancer Therapy. Cancers.

[127] Bu L.-L., Wang H.-Q., Pan Y., Chen L., Wu H., Wu X., Zhao C., Rao L., Liu B., Sun Z.-J. (2021). Gelatinase-Sensitive Nanoparticles Loaded with Photosensitizer and STAT3 Inhibitor for Cancer Photothermal Therapy and Immunotherapy. J. Nanobiotechnol..

[128] Salomon N., Selmi A., Grunwitz C., Kong A., Stanganello E., Neumaier J., Petschenka J., Diken M., Kreiter S., Türeci Ö. (2022). Local Radiotherapy and E7 RNA-LPX Vaccination Show Enhanced Therapeutic Efficacy in Preclinical Models of HPV16+ Cancer. Cancer Immunol. Immunother..

[129] Grunwitz C., Salomon N., Vascotto F., Selmi A., Bukur T., Diken M., Kreiter S., Türeci Ö., Sahin U. (2019). HPV16 RNA-LPX Vaccine Mediates Complete Regression of Aggressively Growing HPV-Positive Mouse Tumors and Establishes Protective T Cell Memory. Oncoimmunology.

[130] Tsai J.M., Nowak R.P., Ebert B.L., Fischer E.S. (2024). Targeted Protein Degradation: From Mechanisms to Clinic. Nat. Rev. Mol. Cell Biol..

[131] Zhong G., Chang X., Xie W., Zhou X. (2024). Targeted Protein Degradation: Advances in Drug Discovery and Clinical Practice. Signal Transduct. Target. Ther..

[132] Zhang C., Liu Y., Li G., Yang Z., Han C., Sun X., Sheng C., Ding K., Rao Y. (2024). Targeting the Undruggables-the Power of Protein Degraders. Sci. Bull..

[133] Kenten J.H., Roberts S.F. (2001). Controlling Protein Levels in Eucaryotic Organisms. US Patent.

[134] Sakamoto K.M., Kim K.B., Kumagai A., Mercurio F., Crews C.M., Deshaies R.J. (2001). Protacs: Chimeric Molecules That Target Proteins to the Skp1-Cullin-F Box Complex for Ubiquitination and Degradation. Proc. Natl. Acad. Sci. USA.

[135] Dang C.V., Reddy E.P., Shokat K.M., Soucek L. (2017). Drugging the “Undruggable” Cancer Targets. Nat. Rev. Cancer.

[136] Li Y., Song J., Zhou P., Zhou J., Xie S. (2022). Targeting Undruggable Transcription Factors with PROTACs: Advances and Perspectives. J. Med. Chem..

[137] Burke M.R., Smith A.R., Zheng G. (2022). Overcoming Cancer Drug Resistance Utilizing PROTAC Technology. Front. Cell Dev. Biol..

[138] Kim H., Park J., Kim J.-M. (2022). Targeted Protein Degradation to Overcome Resistance in Cancer Therapies: PROTAC and N-Degron Pathway. Biomedicines.

[139] Jin J., Wu Y., Zhao Z., Wu Y., Zhou Y.-D., Liu S., Sun Q., Yang G., Lin J., Nagle D.G. (2022). Small-Molecule PROTAC Mediates Targeted Protein Degradation to Treat STAT3-Dependent Epithelial Cancer. JCI Insight.

[140] Mukerjee N., Maitra S., Gorai S., Ghosh A., Alexiou A., Thorat N.D. (2023). Revolutionizing Human Papillomavirus (HPV)-Related Cancer Therapies: Unveiling the Promise of Proteolysis Targeting Chimeras (PROTACs) and Proteolysis Targeting Antibodies (PROTABs) in Cancer Nano-Vaccines. J. Med. Virol..

[141] Zhang S., Lai Y., Pan J., Saeed M., Li S., Zhou H., Jiang X., Gao J., Zhu Y., Yu H. (2024). PROTAC Prodrug-Integrated Nanosensitizer for Potentiating Radiation Therapy of Cancer. Adv. Mater..

[142] Mukerjee N., Mukherjee D. (2025). PROTAC-Based Therapeutics for Targeting HPV Oncoproteins in Head and Neck Cancers. Nano TransMed.

[143] Wang C., Zheng C., Wang H., Zhang L., Liu Z., Xu P. (2022). The State of the Art of PROTAC Technologies for Drug Discovery. Eur. J. Med. Chem..

[144] Li X., Pu W., Zheng Q., Ai M., Chen S., Peng Y. (2022). Proteolysis-Targeting Chimeras (PROTACs) in Cancer Therapy. Mol. Cancer.

[145] Funk A.L., Katerji M., Afifi M., Nyswaner K., Woodroofe C.C., Edwards Z.C., Lindberg E., Bergman K.L., Gough N.R., Rubin M.R. (2025). Targeting c-MYC and Gain-of-Function p53 through Inhibition or Degradation of the Kinase LZK Suppresses the Growth of HNSCC Tumors. Sci. Signal..

[146] Groenland S.L., Mathijssen R.H.J., Beijnen J.H., Huitema A.D.R., Steeghs N. (2019). Individualized Dosing of Oral Targeted Therapies in Oncology Is Crucial in the Era of Precision Medicine. Eur. J. Clin. Pharmacol..

[147] Moreau K., Coen M., Zhang A.X., Pachl F., Castaldi M.P., Dahl G., Boyd H., Scott C., Newham P. (2020). Proteolysis-Targeting Chimeras in Drug Development: A Safety Perspective. Br. J. Pharmacol..

[148] Dietz A., Boehm A., Mozet C., Wichmann G., Giannis A. (2008). Current Aspects of Targeted Therapy in Head and Neck Tumors. Eur. Arch. Otorhinolaryngol..

[149] Smith B.E., Wang S.L., Jaime-Figueroa S., Harbin A., Wang J., Hamman B.D., Crews C.M. (2019). Differential PROTAC Substrate Specificity Dictated by Orientation of Recruited E3 Ligase. Nat. Commun..

[150] Sok J.C., Coppelli F.M., Thomas S.M., Lango M.N., Xi S., Hunt J.L., Freilino M.L., Graner M.W., Wikstrand C.J., Bigner D.D. (2006). Mutant Epidermal Growth Factor Receptor (EGFRvIII) Contributes to Head and Neck Cancer Growth and Resistance to EGFR Targeting. Clin. Cancer Res..

[151] Novoplansky O., Fury M., Prasad M., Yegodayev K., Zorea J., Cohen L., Pelossof R., Cohen L., Katabi N., Cecchi F. (2019). MET Activation Confers Resistance to Cetuximab, and Prevents HER2 and HER3 Upregulation in Head and Neck Cancer: MET/MAPK Drives Resistance to Cetuximab in HNSCC. Int. J. Cancer.

[152] Constantin M., Chifiriuc M.C., Bleotu C., Vrancianu C.O., Cristian R.-E., Bertesteanu S.V., Grigore R., Bertesteanu G. (2024). Molecular Pathways and Targeted Therapies in Head and Neck Cancers Pathogenesis. Front. Oncol..

[153] Yan Y., Kumar A.B., Finnes H., Markovic S.N., Park S., Dronca R.S., Dong H. (2018). Combining Immune Checkpoint Inhibitors with Conventional Cancer Therapy. Front. Immunol..

[154] Kearney V., Chan J.W., Valdes G., Solberg T.D., Yom S.S. (2018). The Application of Artificial Intelligence in the IMRT Planning Process for Head and Neck Cancer. Oral Oncol..

